# Hunger signalling in the olfactory bulb primes exploration, food-seeking and peripheral metabolism

**DOI:** 10.1016/j.molmet.2024.102025

**Published:** 2024-09-03

**Authors:** Romana Stark, Harry Dempsey, Elizabeth Kleeman, Martina Sassi, Sherri Osborne-Lawrence, Sepideh Sheybani-Deloui, Helen J. Rushby, Christen K. Mirth, Karl Austin-Muttitt, Jonathan Mullins, Jeffrey M. Zigman, Jeffrey S. Davies, Zane B. Andrews

**Affiliations:** 1 Monash Biomedicine Discovery Institute and Department of Physiology, Monash University, Clayton, Victoria, Australia; 2 The Florey Institute of Neuroscience and Mental Health, Mental Health Division, Parkville, Melbourne, Australia; 3 Institute of Life Sciences, School of Medicine, Swansea University, Swansea, UK; 4 Center for Hypothalamic Research, Department of Internal Medicine, UT Southwestern Medical Center, Dallas, TX, USA; 5 Division of Endocrinology, Department of Internal Medicine, UT Southwestern Medical Center, Dallas, TX, USA; 6 Department of Psychiatry, UT Southwestern Medical Center, Dallas, TX, USA; 7 School of Biological Sciences, Monash University, Clayton, Victoria, Australia

**Keywords:** Hunger, Ghrelin, Anxiety, Metabolism, Olfaction, Glutamate, Olfactory bulb, Transcriptomics

## Abstract

**Objective:**

Although the metabolic state of an organism affects olfactory function, the precise mechanisms and their impact on behavior and metabolism remain unknown. Here, we assess whether ghrelin receptors (GHSRs) in the olfactory bulb (OB) increase olfactory function and influence foraging behaviors and metabolism.

**Methods:**

We performed a detailed behavioural and metabolic analysis in mice lacking GHSRs in the OB (OB^GHSR^ deletion). We also analsyed OB scRNA-seq and spatial transcriptomic datasets to assess GHSR+ cells in the main and accessory olfactory bulbs, as well as the anterior olfactory nucleus.

**Results:**

OB^GHSR^ deletion affected olfactory discrimination and habituation to both food and non-food odors. Anxiety-like and depression-like behaviors were significantly greater after OB^GHSR^ deletion, whereas exploratory behavior was reduced, with the greatest effect under fasted conditions. OB^GHSR^ deletion impacted feeding behavior as evidenced by altered bout number and duration, as well as buried food-seeking. OB^GHSR^ deletion increased body weight and fat mass, spared fat utilisation on a chow diet and impaired glucose metabolism indicating metabolic dysfunction. Cross referenced analysis of OB scRNA-seq and spatial transcriptomic datasets revealed GHSR+ glutamate neurons in the main and accessory olfactory bulbs, as well as the anterior olfactory nucleus. Ablation of glutamate neurons in the OB reduced ghrelin-induced food finding and phenocopied results seen after OB^GHSR^ deletion.

**Conclusions:**

OB^GHSRs^ help to maintain olfactory function, particularly during hunger, and facilitate behavioral adaptations that optimise food-seeking in anxiogenic environments, priming metabolic pathways in preparation for food consumption.

## Highlights

• OB^GHSR^ deletion impaired olfactory discrimination and habituation to both food and non-food odors.
• OB^GHSR^ deletion increased anxiety-like behaviours and decreased exploratory behaviour.
• OB^GHSR^ deletion caused metabolic dysfunction.
• OB transcriptomic data reveal the majority of OB GHSR+ neurons are glutamatergic.
• Ablation of OB glutamate neurons caused metabolic dysfunction.

## Graphical abstract

**Figure:**
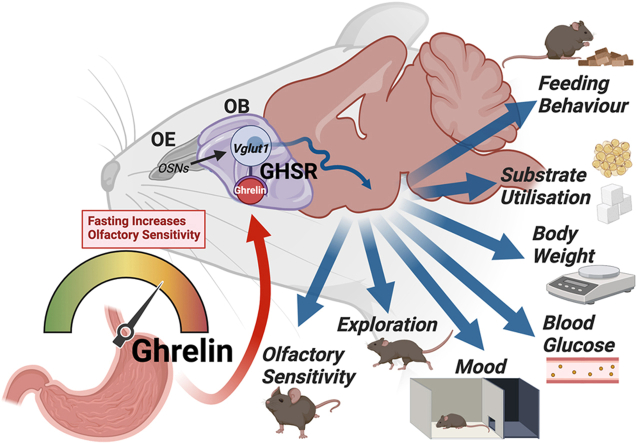

## 1 Introduction

The neural mechanisms that drive appetite and peripheral energy metabolism, such as the balance between fat utilisation and storage, are essential to regulate body weight. Indeed, a diverse range of factors affects these neural mechanisms, including energy need (fasted vs fed), food availability, motivation, emotional valence, reward processing and sensory awareness. Ultimately, the integration of all these factors plays an important role in food-seeking, consumption and peripheral energy metabolism. Although studies have traditionally focused on homeostatic interoceptive mechanisms, largely governed by the hypothalamus and brainstem, recent studies highlight that food consumption and peripheral energy metabolism are not solely dependent on the homeostatic requirements of the body. For example, the integration of external environmental cues, including the visual, auditory or olfactory perception of food availability and palatability, is also important [[1], [2], [3]].

Although sensory information relating to food and food cues comes from a range of sources, olfaction is often the first sensory modality to assess food characteristics and prime behavioral and metabolic responses. In many animals, olfaction plays an essential role in food-seeking [4] and food-related odors stimulate physiologic responses in anticipation of food (salivation, gastric acid secretion, lipid utilisation, blood pressure) [1,3,5,6] to promote behavioral responses that increase appetitive motivation and memory formation [4,7]. Indeed, humans with olfactory dysfunction often report issues with appetite and enjoyment of food, highlighting an olfactory influence over the hedonic appreciation of food [8,9]. Notably, olfactory bulbectomy is a rodent model of depression-like behavior [10].

The influence of olfaction on feeding behavior and metabolism is exemplified by olfactory dysfunction in many metabolic diseases, including diabetes, obesity, and anorexia nervosa [[11], [12], [13]]. Conversely, increased olfactory sensitivity prevented diet-induced obesity in both rodent genetic and pharmacological models [[14], [15], [16]]. In addition, metabolic state regulates olfaction since hunger and energy deprivation increase olfactory discrimination and sensitivity [[16], [17], [18], [19]]. Collectively, these studies point to an important, yet poorly understood, reciprocal relationship between olfaction and metabolism. One potential underpinning mechanism involves the action of circulating metabolic hormones that cross the blood–brain barrier via fenestrated capillaries present in this vascularized region adjacent to the OB [20]. Indeed, a vast array of hormone receptors are found in the OB including receptors for insulin, leptin, cholecystokinin, glucagon-like peptide 1, glucocorticoids and ghrelin [21].

Given that hunger increases olfactory sensitivity, we wanted to assess the mechanisms linking hunger with olfaction and the implications of disrupting this feedback on food intake, metabolism and related mood and foraging behaviors. A possible candidate linking hunger and olfactory function is ghrelin. Ghrelin is a metabolic hormone from the stomach conveying low body energy availability. In response to an energy deficit, ghrelin maximizes energy intake, energy storage, maintains blood glucose and facilitates an optimal behavioral response during energy-seeking in times of metabolic need [22,23]. Ghrelin binds its receptor (Growth Hormone Secretagogue Receptors; GHSR) which is expressed throughout the CNS [24], including the olfactory epithelium and the glomerular, mitral cell, and granular cell layers of the main OB and in the accessory OB [[25], [26], [27], [28]]. Furthermore, the OB has the greatest uptake of radiolabelled plasma ghrelin [29,30] and ghrelin influx across the blood–brain barrier is highest during fasting and lowest in obesity [31]. Ghrelin administration also markedly increases c-fos immunoreactivity in the granular, inner plexiform and mitral cells layers of the OB [32], augments the percentage of c-fos activated OB neurons in response to the odorant 2,3-hexanedione [33] and activates new adult-born OB cells [27]. In humans, ghrelin also enhances food odor conditioning and sniffing [28]. Although these studies highlight how exogenous ghrelin affects olfactory activity, the behavioral and metabolic actions of GHSRs in the OB remain unknown. In this study, we hypothesized that GHSRs in the OB link hunger with olfactory sensitivity to control feeding-related behavior and metabolism.

## 2 Materials and methods

### 2.1 Animals

All experiments were conducted in accordance with the Monash University Animal Ethics Committee guidelines and the Australian code for the care and use of animals for scientific purposes (8th edition) (2013). Male mice were kept under standard laboratory conditions with ad libitum access to food (20% protein 4.8% fat chow diet; Specialty Feeds, Western Australia) and water at 23 °C with a relative humidity of 50–70% in a 12hr light/dark cycle. Mice were group housed (3–4 per cage) to prevent isolation stress unless otherwise stated. Mice were 10–12 weeks at the time of surgery. *Ghsr*-P2A-cre knockin mice were generated via CRISPR genome editing at the Monash Genome Modification Platform, Monash University as a node of Phenomics Australia. A CRISRP/Cas9 approach was used to insert a P2A sequence and Cre recombinase gene immediately upstream of the *Ghsr* translation stop codon at the end of exon 3 of the *Ghsr* gene located on chromosome 3 (MGI:2441906). The sgRNAs used for the insertion were sgRNA1 (5′-TAA CTA CGA GCT GAA ACA GG-′3) cutting 20bp upstream of the *Ghsr* STOP codon and sgRNA2 (5′-TGT GGA GCA ATG AGC GAT GA-3′) cutting 26bp downstream of the *Ghsr* STOP codon. To prevent re-cutting, silent mutations were incorporated in the repair template at the sgRNA1 site and a FRT site was incorporated at the cutting site of sgRNA2, which can also be used to reduce the copy number in case of head to tail insertions. CRISPR-Cas9 sgRNAs (single RNA molecule comprised of both crRNA and tracrRNAsequences) were ordered from Integrated DNA Technologies (Coralville, Iowa, USA). Cas9 nuclease was purchased from Integrated DNA Technologies (Coralville, Iowa, USA; Alt-R® S.p.HiFi Cas9 Nuclease V3) and incubated with sgRNAs to form a ribonucleoprotein (RNP) complex. The ssDNA repair template was generated using the Guide-it Long ssDNA Production System (Takara) according to the manufacturer's instructions. Cas9 nuclease (30 ng/ml), sgRNAs 1 & 2 (30 ng/ml) & ssDNA repair template (30 ng/ml) were microinjected into the pronucleus of C57BL/6J zygotes at the pronuclei stage. Injected zygotes were transferred into the uterus of pseudo-pregnant F1 females. Digital droplet PCR was used to confirm the correct copy number of heterozygous offspring and all genotyping prior to establishing a breeding colony was conducted in-house. To visualise cre-expressing GHSR neurons, heterozygous *Ghsr* Cre mice were crossed with fs-TRAP mice (B6.129S4-*Gt(ROSA)26Sor*^*tm1(CAG-EGFP/Rpl10a,-birA)Wtp*^/J; stock number 022367), which results in the expression of an EGFP-L10a ribosome tag following exposure to Cre recombinase. To assess the role of glutamate neurons in the OB, we used *Vglut1*-ires-cre mice (B6.Cg-*Slc17a7*^*tm1.1(cre)Hze*^/J; stock number #037512, Jackson Laboratory, Maine, USA) injected with Adeno-Associated virus containing cre-dependent expression of caspase (AAV-flex-taCasp3-TEVp, Addgene#45580) into the OB. *Vglut1*-ires-cre mice were crossed with fs-TRAP mice to induce EGFP expression in Vglut1 neurons. These Vglut1xTRAP mice were then injected with AAV-flex-taCasp3-TEVp in the OB to generate Vglut1xTRAP^OB−Caspase^ mice and validate the successful deletion of OB^Vglut1^ neurons.

### 2.2 Surgery

Floxed-GHSR (*Ghsr*^*fl/fl*^) mice were crossed with Ai14 mice (B6.Cg-*Gt(ROSA)26Sor*^*tm14(CAG-tdTomato)Hze*^/J; stock number #007914, Jackson Laboratory, Maine, USA) to develop a *Ghsr*^*fl/fl*^::Ai14 RFP C57/BL6 mouse line. Ai14 mice are used to drive the expression of a red fluorescent protein (tdTomato) in the presence of cre. Targeted deletion of GHSR in the olfactory bulb (OB) was achieved through the bilateral stereotaxic injection of an Adeno-Associated virus containing a cre-recombinase enzyme (AAVpmSyn1-EBFP-Cre, Addgene#51507) into the OB of *Ghsr*^*fl/fl*^::Ai14 RFP (designated OB^GHSR−/−^) and *Ghsr*^*wt/wt*^::Ai14 RFP mice (designated WT) (coordinates: x = ± 0.5, y = 3.2, z = −1.5, −2.7). Mice were given two weeks to recover. Cre-driven RFP expression and Cre expression were confirmed via immunohistochemistry in all experimental mice post-mortem. *Vglut1*-ires-cre mice or Vglut1xTRAP mice were injected with AAV-flex-taCasp3-TEVp using the same coordinates as listed above.

S*urgical procedure:* Mice were anaesthetised using isoflurane at a concentration of 5% for induction followed by 1–3% for maintenance. Anaesthesia was indicated by the loss of the pedal withdrawal reflex, after which mice were positioned in a stereotaxic apparatus. Subcutaneous injection of Metacam (50  μl at 0.25 mg/ml) was performed prior to surgery, and a drop of Lacri-Lube (Allergan) was applied to the eyes to prevent corneal damage during the surgery. An incision was made in the centre of the scalp to expose the bregma on the skull. Two 1-mm diameter holes were drilled in the skull with a drill at the bregma coordinates x = ± 0.5, y = 3.2. A 1 μl Hamilton syringe was then lowered into the brain until reaching z = −2.7 to target the OB. An AAV-BFP-Cre virus (AAVpmSyn1-EBFP-Cre, Addgene#51507) was injected via the syringe into the tissue at a rate of 40 nl/min for 5 min, and repeated at the z coordinate of −1.5, on both sides of the OB. Mice were given 30 min recovery time in a cage placed on a heat pad following surgery and monitored over a two-week period following the surgery. At that time, body weight, appearance, motility observation, and food intake were recorded.

### 2.3 Olfactory tests

*Olfactory habituation test*: This test is used to investigate olfactory detection and discrimination ability. Odors were presented in a sequential pattern with 3 trials per odor, 2 min exposure and 1 min apart. Odors were prepared in Eppendorf tubes with filter paper and five holes. A blank filter paper served as negative control, with a Froot loop (*Kellog's cereal*) as a food-based odor with appetitive value, and rosewater as a novel non-food odor, and were presented in that order, allowing us to broadly assess the role of OB^GHSR^ deletion on olfactory performance. Mice could not directly touch the odor source during the test. All mice were individually habituated to testing cages for 30 min prior to testing. Sniffing time, videoed and recorded by a stopwatch, indicated the level of interest in the odor and sniffing was only scored when the mice had their head oriented directly towards the Eppendorf tube and their nose was within 2 cm of the tube. Mice were tested under fed and 24-hour fasted conditions. Manual behavioral analysis was performed using mutually exclusive behavioral classification with Ethovision version 14.0.1322 (Noldus Information Technology; NL). The percent time of each behavior (walking, stationary, climbing, sniffing, rearing, grooming, digging) was scored in the 1st and 3rd trial in response to the blank filter paper (negative control), Froot loops (food-based odor), and rosewater (non-food odor), equalling 6 individually scored trials per animal.

*Buried Food Finding*: This tests the olfactory performance of mice by measuring how much time is needed to find a buried familiar palatable food (latency); Froot loop (*Kellog's cereal*). All tests were performed 4 h before the start of the dark phase in fed mice, 4-hour short fasted and 24-hour fasted conditions. The mice were given froot loops daily in their cages for three days prior to testing to acclimate them to the scent and minimise neophobia. Mice were placed in standard cages (27 × 18 cm) that mimicked testing conditions (4 cm layer of clean sawdust bedding) the night before each test to minimise stress and novel environment exploration. On the test day, mice were transferred to the testing room and transferred to an empty clean cage for 10 min to acclimatise to the room, while a froot loop was buried approximately 2 cm beneath the surface of the test cage containing the high bedding. The bedding surface was smoothed, and the mouse was then transferred to the test cage. The latency to find the froot loop (e.g. mouse eats or holds the froot loop) was videoed and recorded by an observer standing 2 m away from the cage with a silent stopwatch. A cut-off of 6 min (fasted) or 10 min (fed) was used for the test.

*Three Chamber Preference Test*: This was used to assess olfactory preference. Mice were placed in the central compartment of the three-chamber apparatus containing two empty perforated Eppendorf tubes at opposite chambers and habituated for 10 min. Next, the two Eppendorf tubes were replaced with the other two Eppendorf tubes, containing filter paper, one scented with female urine, the other one a blank scent (6 min). Mice could not directly touch the odor source during the test. To assess the preference for a social stimulus over a non-social stimulus, a similar three-chamber social interaction protocol was used. After the habituation phase with 2 empty wire mesh pencil cups, a social stimulus (female WT mouse) was introduced under one of the cups, while a novel object (scale weight) was introduced under the other cup (test phase 1, 6 min). In a second phase, the object stimulus was then replaced by a novel social stimulus (female WT mouse), and the test mouse was allowed to explore the arena for 6 min (test phase 2). The amount of time spent interacting with either stimulus was recorded.

*Attraction and sensitivity to food and pheromone odours:* Assays were performed with both overnight-fasted and ad libitum-fed mice. Mice were habituated to a new fresh cage for 5 min before the attraction assay where also the preference for one of the cage walls and corners was observed. Then 100 μl of 10% peanut butter in mineral oil (10% w/v) and 100 μl of water were pipetted ∼10 cm from the bottom of the cage and on opposite sides from one another. Mice were allowed to move freely around the cage for 2 min, during which a mounted camera above the cage recorded their movement. The mice were then gently placed back into their home cages and, in case sensitivity to other peanut butter dilutions were measured, the walls of the cage were wiped clean with 80% ethanol and kept for further testing. Time spent near odour and near water was compared between genotypes. To discern if the olfactory ablation had affected sensitivity to odours, we repeated the above assay with four dilutions of peanut butter in mineral oil (1:10, 1:100, 1:1000, and 1:10 000). Additionally, to test for disruptions in sensitivity to pheromone odours, the described assay was performed with three dilutions of female urine (1:10, 1:100, and 1:1000).

### 2.4 Behavioral tests

All tests were performed ∼4 h before the start of the dark phase (14:00) under both fed and overnight fasted conditions. Behavioral experiments, excluding the saccharin preference test, were recorded and analysed using Ethovision version 14.0.1322 (Noldus Information Technology; NL).

*Open Field Test*: This consisted of an open circular arena with a diameter of 80 cm. Within the open field, there was a centre zone and a perimeter zone. Each mouse was initially placed in the same position of the perimeter zone and was then allowed to roam undisturbed in the arena for 5 min.

*Light/Dark Box*: This consisted of two different chambers: a large, illuminated ‘light box’ (48 × 30 cm), and a smaller, closed ‘dark box’ (15 × 30 cm). The chambers were separated by an open arch serving as a passageway. The mice were initially placed in the dark box and were then allowed to roam undisturbed in the apparatus for 5 min.

*Elevated Plus Maze (EPM)*: The EPM consisted of two open arms and two closed arms (5 × 30 cm) that intersected at 90° to form a plus sign with a centre zone (5 × 5 cm) in the middle. It was raised 50 cm from the ground. The mice were initially placed in the central zone facing the north open arm and were then allowed to roam undisturbed through the maze for 5 min.

*Saccharin Preference Test*: This was used to test sensitivity to reward, in which singly housed mice were offered either water or a palatable non-caloric solution of 0.1% Saccharin for 2 h every day (14:00 to 16:00) for 4 days. The preference score was calculated by the saccharin intake (V_s_) normalized to water intake (V_w_): Preference score (%) = V_s_/(V_s_ + V_w_)∗100.

### 2.5 Food intake experiments

Food intake was measured in single-housed mice after a short (4-hour) or 24-hour fast and chow food intake was measured at 1, 2, and 5-hour timepoints after approximately 20 g of chow was reintroduced to the cages manually. For the intraperitoneal (IP) ghrelin-induced feeding experiment, mice were given either a ghrelin injection (0.5 μg per g bodyweight) or a saline (0.9%) injection of the same volume. In addition, mice were housed in an automated feeding monitoring system (BioDaq Feeding Cages, Research Diets, NJ, USA) to measure episodic ad-libitum feeding activity and behavior of singly housed undisturbed mice.

### 2.6 Glucose tolerance test (GTT) and insulin tolerance test (ITT), 2-deoxyglucose challenge

The mice were fasted 4 h before testing in the light phase at 13:00. Fasting blood glucose (t = 0 min) was measured using an ACCU-CHEK blood glucose monitor. For the GTT, glucose was diluted in tap water to a concentration of 0.25 g/ml, and was delivered via oral gavage at a dose of 2 mg/g body weight. The IP insulin dose during the ITT was 0.75 mU/g body weight. 2-Deoxyglucose (2-DG, *Sigma #D6134*) was administered via IP injection at 0.5 mg/g body weight. Blood glucose was measured at 15, 30, 60, 120 min. Blood samples (approx. 20 μl) were collected in tubes containing EDTA at t = 0 min, t = 30 min, and t = 60 min. The course of insulin secretion during the GTT was determined. The counterregulatory response to insulin or 2-DG-induced hypoglycaemia was assessed by measuring corticosterone secretion.

### 2.7 24-Hour fasting blood glucose

After fed blood glucose at t = 0 (09:00) was measured, food was removed from the cages and blood glucose was measured over a 24-hour window as indicated, and then the following day at 09:00. Blood samples (approx. 20 μl) were collected in tubes containing EDTA.

### 2.8 Gastric emptying during the GTT

Fasted mice (4 h) were administered glucose and acetaminophen via an oral gavage dose at 2 g/kg glucose and 0.1 g/kg acetaminophen. Blood glucose and acetaminophen were measured over a 2 h period. Since acetaminophen only enters the bloodstream after it passes the stomach, its appearance in blood can be used as an estimate for gastric emptying. Plasma acetaminophen was measured via colorimetric detection kit (2k99-20, Abbott).

### 2.9 Faeces triglycerides

Faecal lipids were extracted using the principle of a Folch Extraction protocol. In brief, faecal samples over a 24 h period were collected and total faecal weight per day recorded. 100 mg of faeces was used for analysis, first softened in 400 μl ddH_2_O, then 1.5 ml of cold chloroform:methanol (2:1) mixture added and lysed using a Qiagen bead TissueLyser, mixed at RT for 20 min prior to centrifugation for 30 min at 9000 rpm. The lower liquid phase was transferred to a fresh tube containing 200 μl 0.9% NaCl, centrifuged for 5 min at 2000 rpm. The lower organic phase was transferred into 40 μl of chloroform:Triton-X (1:1) solution, dried with a speedvac overnight. To measure lipids, 200 μl of ddH_2_O was added to the remaining triton-lipid solution and a TG (Sigma) or NEFA assay kit (Novachem) was used.

### 2.10 Indirect calorimetry

Mice were placed in the Promethion system (Sable Systems International) 2–3 weeks after deletion of OB^GHSRs^ and prior to significant weight gain. Metabolic parameters including oxygen consumption (VO2; ml/hr), carbon dioxide production (VCO2; ml/hr), respiratory exchange ratio (RER), energy expenditure (EE; kcal/hr), and locomotor activity were measured. Mice were single-housed and had ad libitum access to food and water in overhead feeders attached to electronic balances to measure food and water intake. No data were acquired in the respiratory cages for 5 days to permit acclimation to their new environment. Baseline metabolism was assessed within a 48-hour period.

### 2.11 Hormone level analyses

Plasma insulin concentrations were determined using an ELISA (CrystalChem #90080) according to manufacturer's instructions. Corticosterone concentrations were determined using an ELISA (Abcam #ab108821) according to manufacturer's instructions.

### 2.12 PCR and primers

Total RNA was extracted using a guanidium-phenol-chloroform phase separation method, according to the Qiazol® Lysis Reagent (Qiagen #79306) manufacturer's protocol, followed by precipitation of the RNA pellet using isopropanol, 10 min incubation at room temperature and pelleted at 12 000×*g*, 4 °C, 10 min. The pellet was washed 3 times with 75% ethanol prior to reconstitution in RNAfree water. The relative purity and concentration of the RNA was determined using a QIAEXPERT spectrophotometer. Total RNA was treated with DNase I (Qiagen #79254), and complementary DNA (cDNA) was synthesized from RNA using iScript cDNA Synthesis Kit (Bio-Rad #1708891). For qPCR, Fast SybrGreen (Thermofisher Scientific #4385617) was used for amplification and detection, with 25 ng cDNA loaded per well. The Rotor-Gene Q real-time PCR cycler (Qiagen) was used to determine the mRNA expression level for the genes of interest. Following validated primers were used: GHSR: Forward 5′- GCTGCTCACCGTGATGGTAT-3′, Reverse 5′-ACCACAGCAAGCATCTTCACT-3′, Actin: Forward 5′-CCAGATCATGTTTGAGACCTTC-3′, Reverse: 5′-CATGAGGTAGTCTGTCAGGTCC-3′.

### 2.13 Western blot

Proteins were detected by Western blot analysis after separating 30–50 μg of total protein lysate prepared in RIPA buffer with protease inhibitor cocktail (cOmplete™, Roche) on a 4–15% Mini-PROTEAN® TGX™ Precast Gel (BioRad) and transferring to a polyvinylidene difluoride membrane (Immobilon-P 0.45 μm, Millipore). The blots were blocked for 1 h with 3% BSA and then probed with GHSR Polyclonal Antibody (ThermoFisher # PA5-28752, 1:1000 dilution) or anti-Actin (Sigma #A2066) after stripping of the membrane and detected via enhanced chemiluminescence (ECL, Sigma). Westerns were analysed using ImageLab Software (BioRad). A reference sample was included in all blots and GHSR protein expression was normalized to actin.

### 2.14 Immunohistochemistry

To confirm viral injection, animals were deeply anesthetized with isoflurane and perfused with 0.05 M PBS, followed by 4% paraformaldehyde. Brains were postfixed in 4% paraformaldehyde overnight at 4 °C, then placed in 30% sucrose. Brains were cut at 40 μm on a cryostat, and every fourth section was collected and stored in cryoprotectant at −20 °C. Sections were washed in 0.1 M phosphate buffer (PB), incubated with 1% hydrogen peroxide (H_2_O_2_) for 15 min to quench endogenous peroxidase activity, and blocked for 1 h with 5% normal horse serum (NHS) in 0.3% Triton in 0.1 M PB. Sections were incubated with rabbit anti-DsRed (TaKaRa Bio, cat. no. 632496) at 1:1000 and guinea pig anti-Cre (Synaptic Systems, cat. No. 257 004) in diluent of 5% NHS in 0.3% Triton in 0.1 M PB. After incubation, the sections were washed and incubated with Alexa Fluor 594 donkey anti-rabbit antibody and Alexa Fluor 488 anti-guinea pig (ThermoFisher) at 1:2000 in 0.3% Triton in 0.1 M PB. Sections were then washed, mounted, and coverslipped.

### 2.15 Bioinformatics analysis

To confirm the expression of *Ghsr* in the olfactory bulb, the Mouse Whole Brain Atlas database [34] was searched programmatically using a Python notebook. The “OLF” (olfactory area) microdissected region of the scRNA-seq dataset was filtered to cells with non-zero Ghsr expression. Each cell is classified with a cluster-type from the WMB-taxonomy resource. Because the “OLF” region is larger than just the main and accessory olfactory bulbs, the results were refined via cross-reference with the MERFISH spatial transcriptomics (ST) dataset. Each ST cell is classified with both a WMB-taxonomy cluster-type and a localisation to an anatomical structure in the Allen CCFv3 map [35]. The ST cells belonging to relevant cluster-types were retrieved from slides 60, 62, 64, 66, and 67 (Figure 8B). For each cell cluster-type, the fraction of cells of that type in the scRNA-seq data expressing Ghsr was calculated. Using this, the expected numbers of Ghsr+ cells in the ST data were imputed for each relevant anatomical structure: the MOB (main olfactory bulb), AOB (accessory olfactory bulb), and AON (anterior olfactory nucleus).

### 2.16 Statistical analysis

Statistical analyses were performed using GraphPad Prism for MacOS X. Data are represented as mean ± SEM. Two-way ANOVAs with post hoc tests were used to determine statistical significance. A two-tailed Student's paired or unpaired t-test (see figure legends for specific details) was used when comparing genotype only. p < 0.05 was considered statistically significant and is indicated on figures and in figure legends.

## 3 Results

### 3.1 Deletion of OB^GHSR^

**Figure 1:**
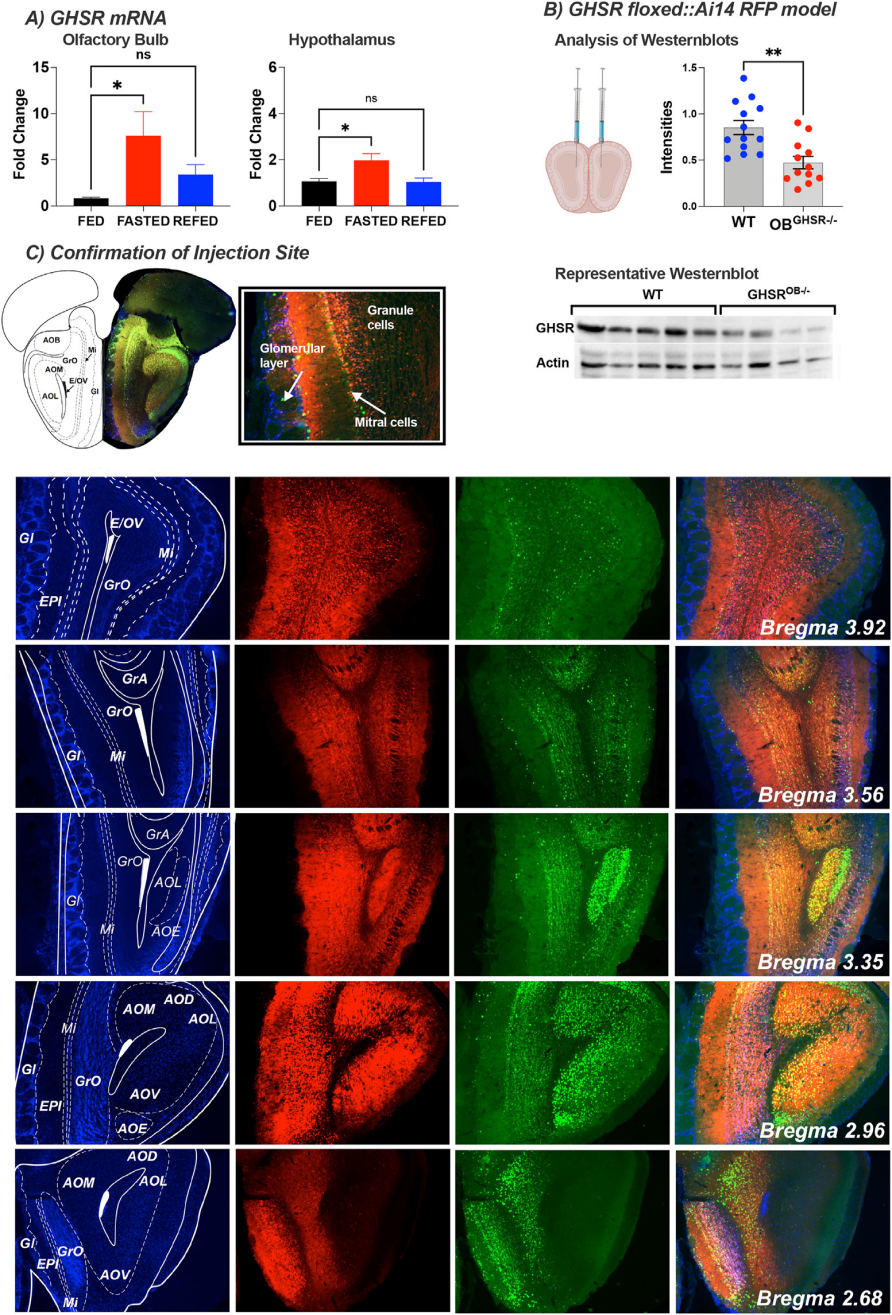
**GHSR expression in the olfactory bulb during different metabolic conditions and after deletion. A)** GHSR mRNA expression in the olfactory bulb and hypothalamus changes during fed (n = 7/7), fasted (n = 11/15) and refed (n = 5/6) conditions. **B)** Confirmation of GHSR deletion in the olfactory bulb; schematic created with BioRender.com. Deletion efficacy after viral Cre recombination in the GHSR floxed::Ai14 RFP model was confirmed by Western blot analysis. Protein GHSR expression intensities of 3 blots (top, WT = 13, OB^GHSR−/−^ = 12) were normalized to corresponding actin. Representative blot (below). **C)** Immunohistochemistry for confirmation of injection site. Cre recombinase immunoreactivity (green) and cre-mediated expression of red fluorescence protein (RFP, red) was used to visualise the viral spread in the olfactory bulb ranging from bregma 4.28 to 2.68, in both OB^GHSR−/−^ and WT respectively. *AOD anterior olfactory nucleus, dorsal part; AOE anterior olfactory nucleus, external part; AOL anterior olfactory nucleus, lateral part; AOM anterior olfactory nucleus, medial part; AOV anterior olfactory nucleus, ventral part; EPI external plexiform layer of the olfactory bulb; E/OV ependymal and subendymal layer/olfactory ventricle; Gl glomerular layer; GrA granular cell layer of the accessory olfactory bulb; GrO granular cell layer of the olfactory bulb; Mi mitral cell layer of the olfactory bulb.* Data are presented as mean +/− SEM. All specific statistical information is reported in supplementary table 1.

**Suppl. Figure 1:**
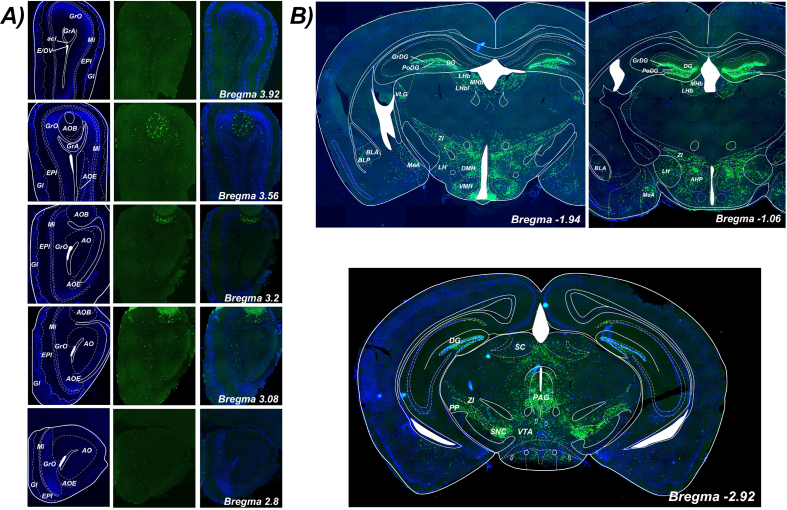
GHSR-expressing cells in the brain of GHSR-p2A-cre x TRAP transgenic mice. A) GHSR expressing neurons in OB of GHSR x TRAP transgenic mice. EGFP-labelled neurons are found in the main olfactory and accessory olfactory bulbs but not the AON. *aci anterior commissure, intrabulbar part; AO anterior olfactory nucleus; AOB accessory olfactory bulb; AOE anterior olfactory nucleus, external part; AOM anterior olfactory nucleus, medial part; EPI external plexiform layer of the olfactory bulb; E/OV ependymal and subendymal layer/olfactory ventricle; Gl glomerular layer; GrA granular cell layer of the accessory olfactory bulb; GrO granular cell layer of the olfactory bulb; Mi mitral cell layer of the olfactory bulb. B)* GHSR expression (green) in various brain regions, especially high expression in the DG of the hippocampus, hypothalamus and midbrain. *AHP anterior hypothalamic area, posterior part; BLA basolateral amygdaloid nucleus, anterior part; BLP basolateral amygdaloid nucleus, posterior part, DG dentate gyrus; DMH dorsomedial hypothalamus; GrDG granular layer of the dentate gyrus; LH lateral hypothalamic area; LHb lateral habenular nucleus; LHbl lateral habenular nucleus, lateral part; MeA medial amygdaloid nucleus, anterior part; MHb medial habenular nucleus; PAG periaqueductal gray; PoDG polymorph layer of the dentate gyrus; PP peripeduncular nucleus; SC superior colliculus; SNC substantia nigra, compact part; VLG ventral lateral geniculate nucleus; VMH ventromedial hypothalamic nucleus; VTA ventral tegmental area; Zl zona incerta.*

We first determined the effects of a 14-hr overnight fast and 2 h of re-feeding on GHSR mRNA expression in the OB (OB^GHSR^) and hypothalamus of C57Bl6 mice. Fasted mice exhibited 7.5-fold and 2-fold increases in GHSR expression in the OB and hypothalamus, respectively, over that observed in ad lib-fed mice (Figure 1A). Thus, hunger regulates OB^GHSR^ gene expression, highlighting it may be an important target to manipulate hunger signaling in the OB. To assess the role of OB^GHSR^ in feeding-related behavior and metabolism, we generated a temporal and OB-specific deletion of GHSRs (OB^GHSR−/−^) in adult mice using bilateral stereotaxic injections of AAV-cre into *Ghsr*^*fl/fl*^::Ai14 RFP (OB^GHSR−/−^) and *Ghsr*^*wt/wt*^::Ai14 RFP mice (WT) mice. GHSR protein expression in the OB (from bregma 4.0 to bregma 2.6) was significantly reduced in OB^GHSR−/−^ compared to WT controls (Figure 1B), confirming the successful knockdown of GHSRs in the OB. To visualise the spread of cre recombinase expression in the OB following stereotaxic surgery we used immunohistochemistry for cre recombinase and red fluorescence protein (RFP). Expression of RFP is dependent on cre expression and is used as a secondary approach to visualize viral spread. Cre recombinase immunoreactivity and cre-induced expression of RFP was observed in main olfactory bulb (MOB) in both the granular and mitral cell layer, anterior olfactory nucleus (AON) and accessory olfactory bulb (AOB) ranging from bregma 4.28 to 2.68, in both OB^GHSR−/−^ and WT mice, respectively (Figure 1C). Although, the expression of cre recombinase and RFP confirms the accuracy of injections to broadly cover the OB, OB^GHSR^ deletion only occurs in regions expressing cre recombinase and GHSR. To assess the location of GHSRs in the olfactory-related areas, we generated a *Ghsr*-P2A-cre mouse line and crossed this with fs-TRAP mice, which resulted in the expression of an EGFP-L10a ribosome tag following exposure to cre recombinase. Our studies demonstrated EGFP-labelled GHSR cells in the granular and mitral cell layers in the MOB and AOB but not the AON (Suppl Figure 1A). Thus, despite the high expression of cre recombinase in the AON, deletion of GHSRs is specific to the MOB and AOB, which we refer as OB^GHSR^ deletion. Moreover, we observed extensive EGFP-labelled GHSR neurons in the hypothalamus, hippocampus, amygdala and midbrain (Suppl Figure 1B), consistent with previous reports of GHSR localisation [24,25].

### 3.2 OB^GHSR^ affects olfaction

**Figure 2:**
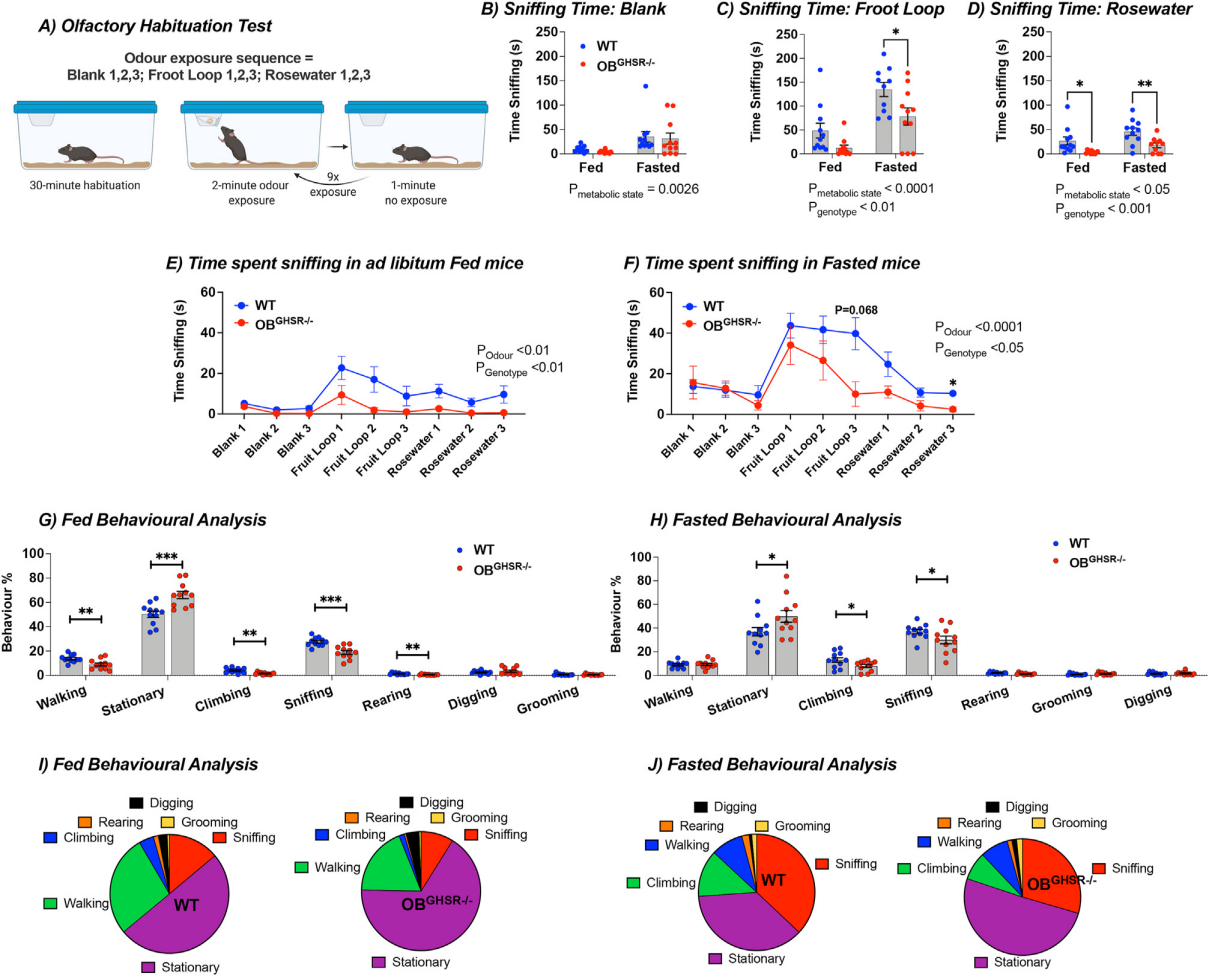
**Reduced olfactory performance after OB**^**GHSR**^**deletion in olfactory habituation test. A)** Schematic of the olfactory habituation task created with BioRender.com. Mice were acclimatised to testing conditions for 30 min, then exposed to an odor for 3 consecutive times for 2 min each separated by 1 min (WT = 11, OB^GHSR−/−^ = 11). Odor was presented on filter paper inside an Eppendorf tube with a perforated lid. **B-D)** Sniffing time fed versus fasted (**B**) Blank (Two-way ANOVA, P_metabolic state_ = 0.0026, P_genotype_ = 0.5631), (**C**) Froot Loop (Two-way ANOVA, P_metabolic state_<0.0001, P_genotype_ = 0.0024), (**D**) Rosewater (Two-way ANOVA, P_metabolic state_ = 0.0145, P_genotype_ = 0.0005). **E-F)** Time spent sniffing in (**E**) ad libitum fed condition across the 3 different odor exposures (Two-way ANOVA, P_odor_ = 0.0026, P_genotype_ = 0.0036), versus (**F**) fasted condition (Two-way ANOVA, P_odor_<0.0001, P_genotype_ = 0.0482, P_odor∗genotype_ = 0.0595). **G-H)** Behavioral analysis during the olfactory habituation test in (**G**) fed, versus (**H**) fasted condition, combined for foot loop and rosewater trials (6 trials per mouse). Behavioral differences were observed in exploratory behaviors, such as walking, stationary, climbing, sniffing and rearing behaviors in the (**G**) fed state (P = 0.0052, P = 0.0007, P < 0.0015, P = 0.0002, P < 0.004), as well as for stationary, climbing and rearing behavior in the (H) fasted state (P = 0.047, P = 0.047, P = 0.048; n = WT = 11, OB^GHSR−/−^ = 11). **I-J)** Pie charts of behaviors (walking, climbing, rearing, digging, grooming, sniffing, stationary) during (**I**) fed, and (**J**) fasted conditions. Data are presented as mean +/− SEM. All specific statistical information is reported in supplementary table 1.

**Figure 3:**
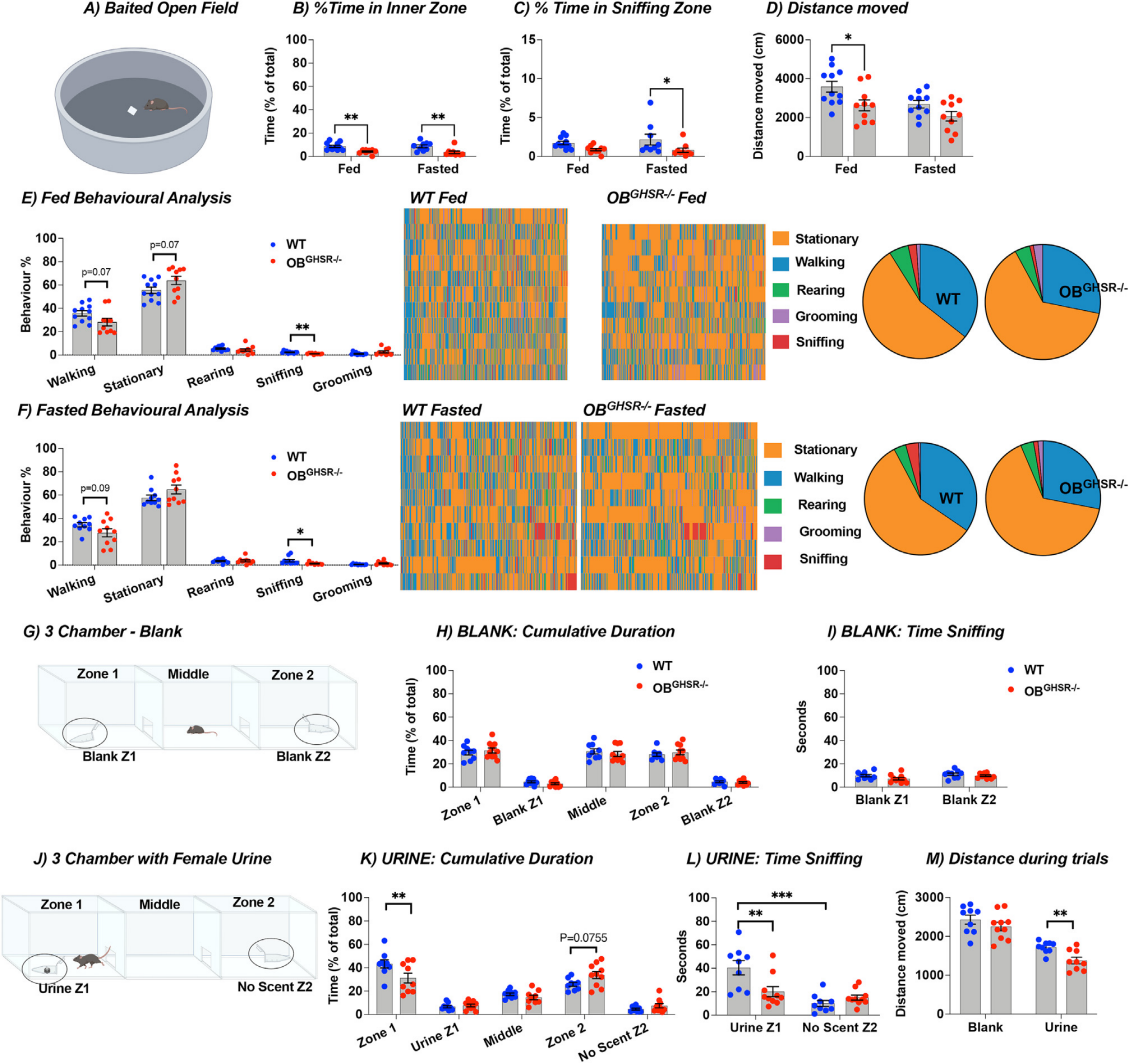
**OB**^**GHSR**^**deletion reduced interest in food-related and social odors A)** Schematic of the baited open field test created with BioRender.com. A peanut butter-scented filter paper was placed in the middle of an open field arena, and mice were allowed to roam undisturbed in fed versus fasted conditions (WT = 11, OB^GHSR−/−^ = 10). **B-D)** Percent Time in Inner Zone (**B**), and in sniffing zone (**C**), with the distance moved (D). OB^GHSR−/−^ mice spend less time in the inner zone (Two-way ANOVA, P_genotype_<0.0001), and move less (Two-way ANOVA, P_genotype_ = 0.0039). **E,F)** Behavioral analysis with pie charts of behaviors (stationary, walking, rearing, grooming, sniffing) in fed (**E**) and fasted (**F**) conditions. Behavioral analysis during the baited open field task reveals that OB^GHSR−/−^ mice have less interest in active behaviors and spent more time stationary (multiple unpaired t tests). **G)** Schematic of the 3 chamber exploration with no scent or female urine on filter paper inside an Eppendorf tube with a perforated lid created with BioRender.com (WT = 9, OB^GHSR−/−^ = 10). **H, I**) Cumulative duration (**H**) and time sniffing (**I**) in the different zones with no scent (blank) present. **K,L)** Cumulative duration (**K**) and time sniffing (**L**) in the different zones when mice were presented with female urine. **M)** Distance during trials. While there was no difference in the exploration task when no scent was present, OB^GHSR−/−^ mice spent significantly less time sniffing the female urine (Two-way ANOVA, P_genotype∗zone_ = 0.0008) and explore less (multiple unpaired t tests, P_urine_ = 0.002697). Data are presented as mean +/− SEM. All specific statistical information is reported in supplementary table 1.

**Suppl. Figure 2:**
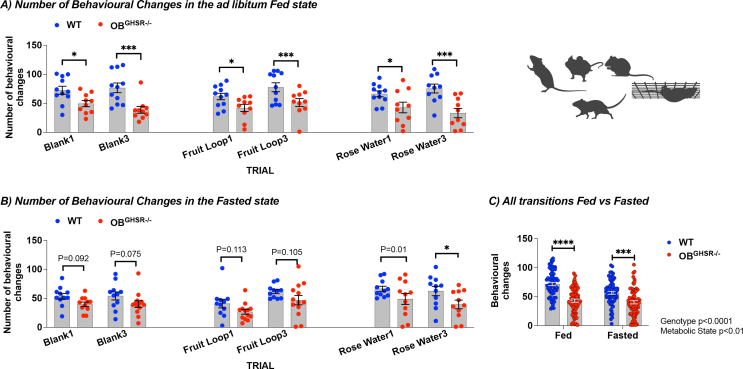
Olfactory habituation test. A) Number of behavioral changes in ad libitum fed condition, schematic created with BioRender.com. B) Number of behavioral changes in fasted condition. C) Number of behavioral changes during fed and fasted conditions. OB^GHSR−/−^ mice exhibited fewer behavioral changes during the olfactory habituation test, suggesting less engagement and behavioral flexibility in exploratory activities (Two-way ANOVA). Data are presented as mean +/− SEM. All specific statistical information is reported in supplementary table 1.

**Suppl. Figure 3:**
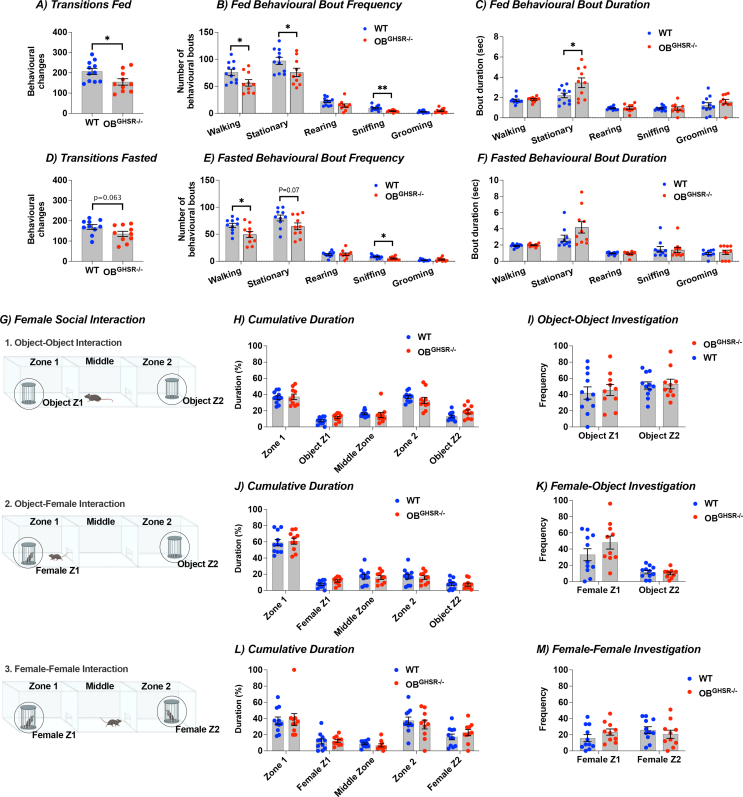
Baited open field and female social interaction test. A) Number of behavioral changes in fed condition. B) Behavioral bout frequency in fed condition. C) Time spent in a behavioral bout during fed condition. D) Number of behavioral changes in fasted condition. E) Behavioral bout frequency in fasted condition. F) Time spent in a behavioral bout during fasted condition. OB^GHSR−/−^ mice show different behavioral patterns with more stationary behavior and less involvement in sniffing and walking exploration or behavioral transitions (Students t-test). G-M) A schematic of a female social interaction test using a 3-chamber preference protocol created with BioRender.com, where mice either explore the 3 chambers with 2 empty wire mesh pencil cups, or a social stimulus (female WT mouse) under one or both cups. The amount of time spent interacting with either stimulus was recorded. H,I) Object–Object Interaction, 2 empty wire cups. J,K) Object–Female Interaction. L,M) Female–Female Interaction. No differences in the duration spent in the different zones nor frequencies of investigations between the groups were observed (Two-way ANOVA). Data are presented as mean +/− SEM. All specific statistical information is reported in supplementary table 1.

To assess whether OB^GHSR^ deletion affects olfactory function, we used various olfactory performance tests in fed and overnight fasted conditions. Importantly, we used olfactory tests based on sniffing, as a broad index of olfactory function [36], because this simultaneously allowed for an accurate and detailed assessment of behavior during each test. In an olfactory habituation test (Figure 2A), OB^GHSR^ deletion significantly reduced sniffing time to froot loops and rosewater. Although fasting increased sniffing time compared to fed mice in both groups, this was still significantly attenuated in OB^GHSR−/−^ mice (Figure 2B–F). Video analysis of behavior during the olfactory habituation test revealed that OB^GHSR−/−^ mice spent less time in active states, such as walking, climbing, rearing and more time in stationary behavior over the entire testing period (6 individual odor exposure trials per animal were scored; Figure 2G–J). Furthermore, OB^GHSR−/−^ mice exhibited fewer behavioral changes during the olfactory habituation test (Suppl Figure 2), suggesting less engagement and behavioral flexibility in exploratory activities. This was similar in an odor-baited field test, in which OB^GHSR−/−^ mice spent less time sniffing peanut butter scented paper in an open field environment (Figure 3A–D). Although time spent in the inner zone was significantly reduced in both fed and fasted OB^GHSR−/−^ mice, only fasted OB^GHSR−/−^ mice spent less time in the sniffing zone (Figure 3C). Behavioral analysis during the baited open field test revealed that OB^GHSR−/−^ mice show different behavioral patterns with more stationary behavior and less involvement in sniffing and walking exploration (Figure 3E–F) or behavioral transitions (Suppl Figure 3A-F).

Since olfaction is important for social behavior, we used a three-chamber preference protocol to assess interest in non food-related social olfactory stimuli (Figure 3G-M). Although time spent sniffing blank tubes in the habituation phase did not differ between control and experimental groups (Figure 3H–I), OB^GHSR−/−^ mice spent less time in the chamber with the female mouse urine scent and sniffed less (Figure 3K-L). Distance moved was significantly lower in OB^GHSR−/−^ mice in the chamber paired with urine scent (Figure 3M). A similar protocol was used to investigate social interaction by introducing a female mouse under a mesh cup versus a novel object (test phase 1) or subsequently a novel female mouse (test phase 2). No difference between the groups were observed (Suppl Figure 3G-M), suggesting the additional visual cues made up for the lack of olfactory deficits. Collectively, these experiments demonstrate OB^GHSR^ deletion has a profound effect to decrease olfactory function, assessed primarily by sniffing [36] and behavior, to both food and female urine.

### 3.3 OB^GHSR^ regulate exploratory activity and mood

**Figure 4:**
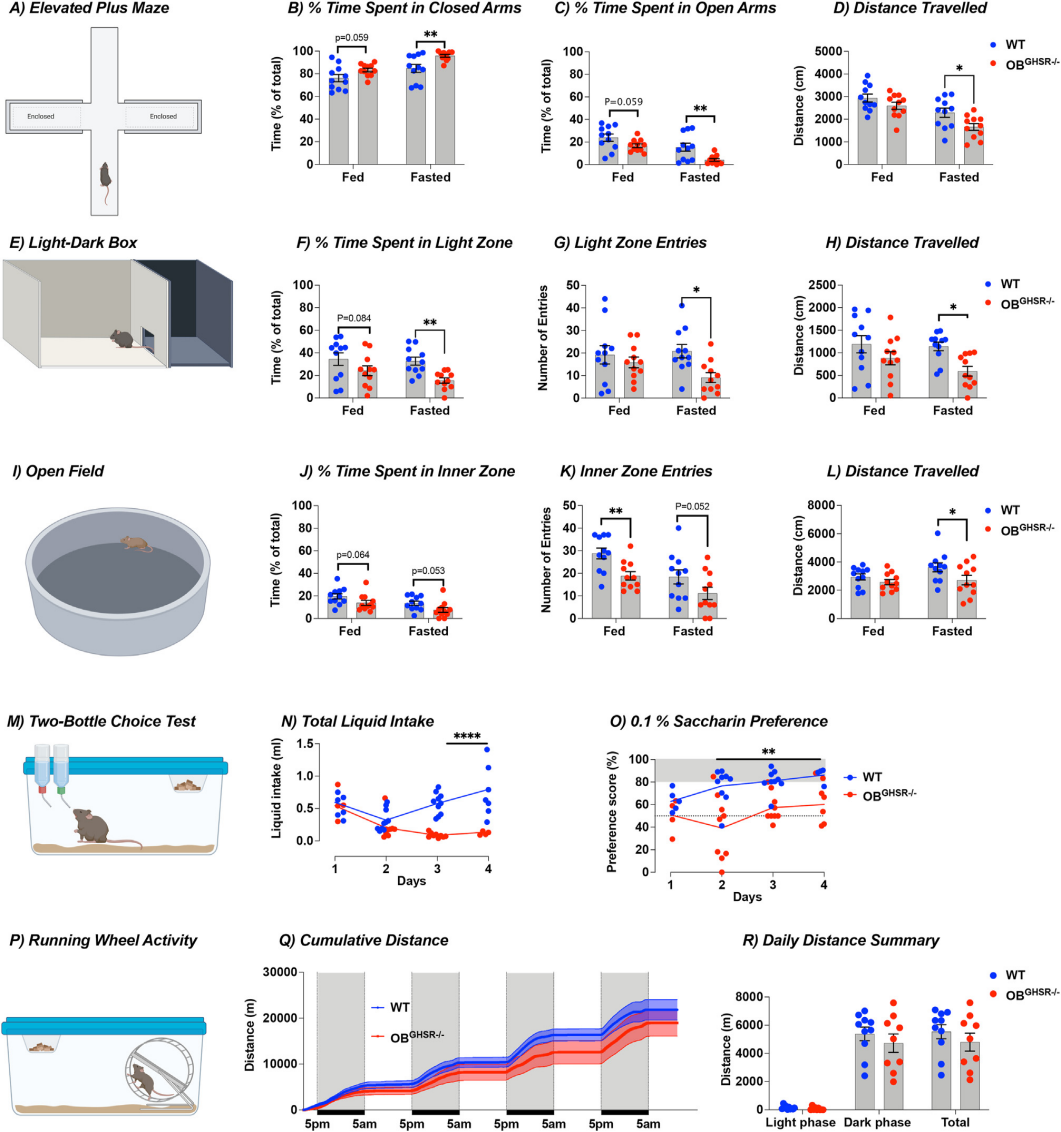
**OB**^**GHSR**^**deletion affects mood, anxiety, and hedonia. A-D)** Schematic of the elevated plus maze with 2 open arms and 2 closed arms in fed and fasted conditions created with BioRender.com (WT = 11, OB^GHSR−/−^ = 11). Time spent in the more anxiogenic open zone was measured in fed versus fasted conditions. While mice with GHSR deletion spent more time in closed arms (**B**) and less time in open arms (**C**), this was significant in the fasted state, where mice also moved less (**D**) (Two-way ANOVA). **E-H)** Schematic of the Light-Dark Box in fed and fasted conditions created with BioRender.com (WT = 11, OB^GHSR−/−^ = 11). Percent time in the anxiogenic light zone (**F**) and light zone entries (**G**) were significantly lower in OB^GHSR−/−^ mice when fasted, also with less distance travelled (**H**) during fasted conditions (Two-way ANOVA). **I-L)** Schematic of the Open Field Test in fed and fasted conditions created with BioRender.com (WT = 11, OB^GHSR−/−^ = 11). Percent time (**J**) in the anxiogenic inner zone tended to be decreased in both metabolic states with fewer inner zone entries (**K**), and less distance travelled (**L**) (Two-way ANOVA). **M-O**). Schematic of the Two-Bottle Choice Test created with BioRender.com. Mice were offered either water or a palatable non-caloric 0.1% Saccharin solution for 2 h every day (WT = 10, OB^GHSR−/−^ = 9). OB^GHSR−/−^ mice have a significantly lower saccharin intake (**N**) and calculated saccharin preference score (**O**) (Two-way ANOVA). **P–R).** Schematic of the home cage running wheel activity created with BioRender.com. Cumulative distance (**Q**) and daily distance summary (R) for 4 days show no difference between the genotypes (multiple unpaired t tests). Data are presented as mean +/− SEM. All specific statistical information is reported in supplementary table 1.

In the olfactory experiments described above we observed numerous indicators of impaired exploratory behavior in OB^GHSR−/−^ mice, suggesting potentially increased anxiety-like behavior. To further investigate anxiety-like behavior in OB^GHSR−/−^ mice, we assessed behavior using elevated plus maze (Figure 4A), a light–dark box (Figure 4E), and the open field (Figure 4I) tests. In the elevated plus maze OB^GHSR−/−^ mice spent more time in the closed arms and moved less in the fasted state (Figure 4B–D). This was similar for the light–dark box, where OB^GHSR−/−^ mice spent less time in the light zone with fewer light zone entries and moved less in the fasted state (Figure 4F–H). Similarly, in the open field arena, fasted OB^GHSR−/−^ mice spent less time in the inner zone, with fewer inner zone entries and moved less (Figure 4J-L). Of note, the differences in anxiety-like behaviors were not due to a generalized impairment in locomotor activity measured using home-cage running wheels (Figure 4P-R), or home-cage activity assessed in metabolic cages (Figure 6I,J). The deficit in locomotor activity in OB^GHSR−/−^ mice was specific to the stressful acute effects of behavioural testing in unfamiliar environments. Anxiety-like behavior is often linked with depression-like behaviors in mice [37,38] and OB bulbectomy is a rodent model of depression [10], therefore we used a saccharin preference test of anhedonia to assess depression-like behavior. Anhedonia is the inability to experience pleasure and we observed that OB^GHSR−/−^ mice consumed less saccharin over 4 days (Figure 4M−O). Taken together, these results suggest that the OB^GHSR^ deletion results in greater depression-like and anxiety-like behavior. The greater number of post hoc differences were observed only in the fasted state, suggesting the effect on anxiety-like behavior is more pronounced in the fasted state.

### 3.4 OB^GHSR^ regulate food-seeking but not food intake

**Figure 5:**
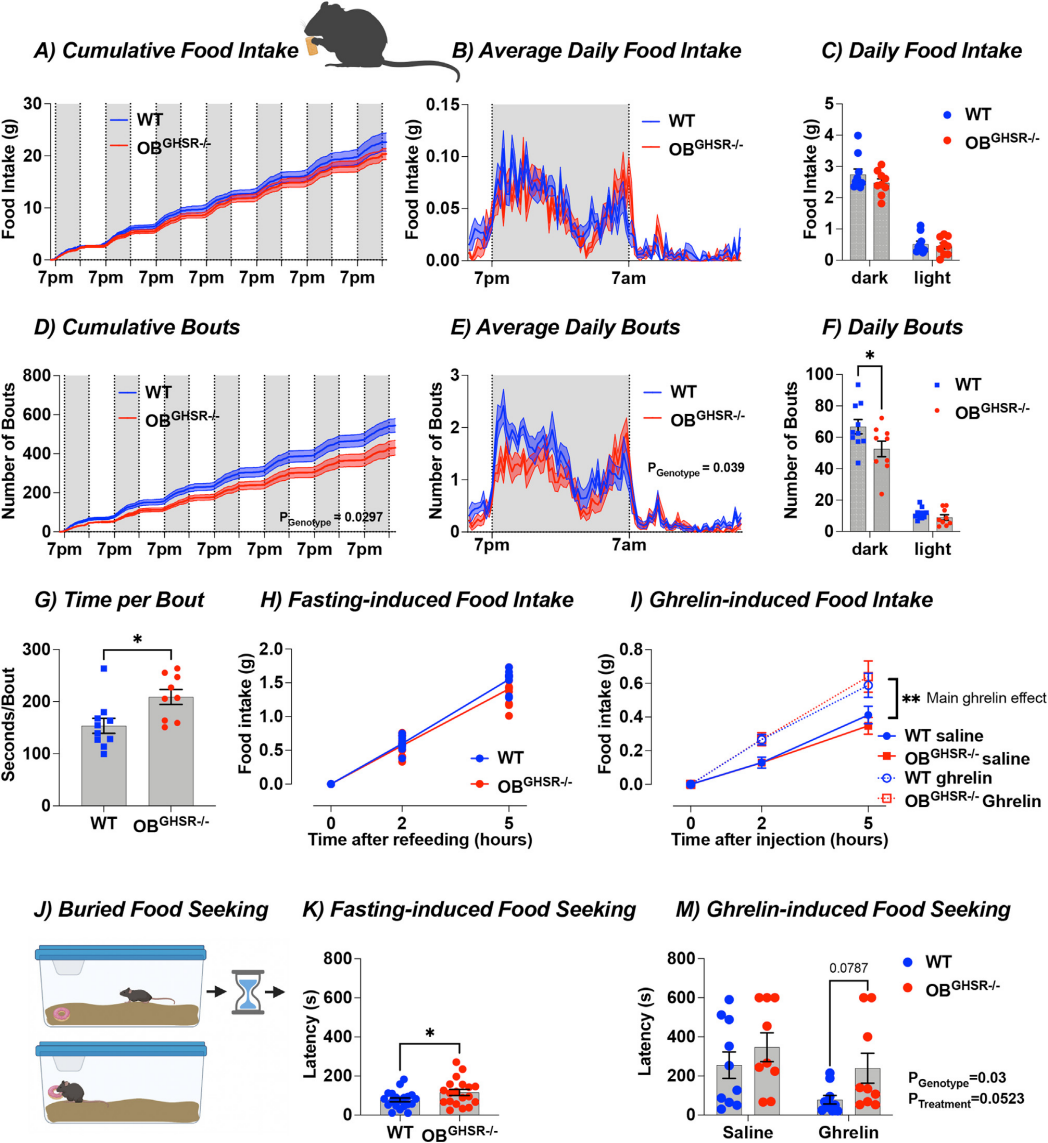
**OB**^**GHSR**^**deletion does not affect food intake but feeding behavior and food seeking. A-G)** Food intake in BioDaq Feeding Cages (WT = 11, OB^GHSR−/−^ = 11). Cumulative Food Intake (**A**), with average daily food intake (**B**), and daily food intake during the dark and light phase (**C**) during a 7-day period. Average food intake was not different between the genotypes (Two-way ANOVA). However, ingestive behavior was different between the groups as shown with cumulative bouts (**D**), average daily bouts (**E**), daily bouts (**F**), and time spent per bout (**G**). OB^GHSR−/−^ mice had significantly fewer feeding bouts (Two-way ANOVA) and spent more time per bout (Two-tailed unpaired t test, P = 0.0155). **H)** Fasting-induced food intake (WT = 11, OB^GHSR−/−^ = 11). Food intake in WT and OB^GHSR−/−^ following an overnight fast 2 h and 5 h after the food was reintroduced. There was no significant different between the groups (Two-way ANOVA). **I)** Ghrelin-induced food intake (WT_saline_ = 18, WT_ghrelin_ = 20, OB^GHSR−/−^_saline_ = 21, OB^GHSR−/−^_ghrelin_ = 20). Food intake in WT and OB^GHSR−/−^ following intraperitoneal injection of ghrelin (0.5 μg/g). While we observed a significant effect of ghrelin on food intake, there was no difference between the two genotypes (Two-way ANOVA). **J-L)** Schematic of the Buried Food Seeking Test created with BioRender.com, which records the time needed to find familiar palatable food (froot loop) buried in high bedding. **K)** Fasting-induce food-seeking after a 4- hour fast (WT = 20, OB^GHSR−/−^ = 19). OB^GHSR−/−^ mice took significantly longer time to find the froot loop (Two-tailed unpaired t test, P = 0.0428). **L)** Ghrelin-induced food seeking (WT_saline_ = 10, WT_ghrelin_ = 10, OB^GHSR−/−^_saline_ = 9, OB^GHSR−/−^_ghrelin_ = 9). OB^GHSR−/−^ mice needed more time to find the froot loop (Two-way ANOVA, P_genotype_ = 0.03). Data are presented as mean +/− SEM. All specific statistical information is reported in supplementary table 1.

**Suppl. Figure 4:**
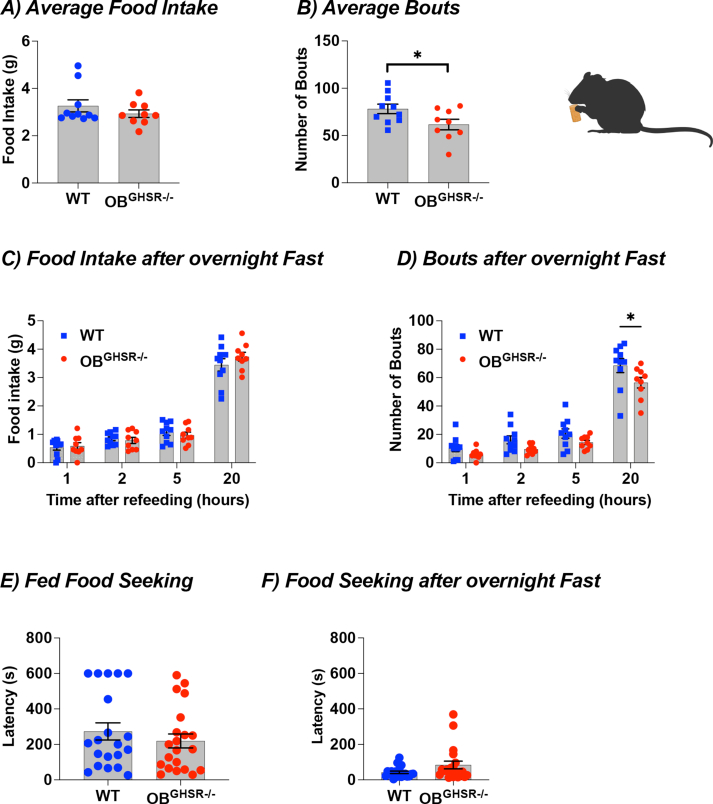
Feeding behavior. A-D) Feeding behavior in BioDaq feeding cages (schematic created with BioRender.com; WT = 10, OB^GHSR−/−^ = 9). A) Average Food Intake. B) Average Bouts. C) Food intake after overnight fasting. D) Bouts after overnight fasting. E) Food seeking in ad libitum fed conditions. F) Food seeking after overnight fasting (Student's t-tests and two-way ANOVAs). Data are presented as mean +/− SEM. All specific statistical information is reported in supplementary table 1.

Since ghrelin administration increases food intake, we performed feeding experiments to investigate the effects of OB^GHSR^ deletion on food seeking and consumption. Although total cumulative food intake was not significantly different (Figure 5A–C, Suppl Figure 4A), OB^GHSR−/−^ mice displayed significant differences in feeding behavior with fewer feeding bouts during the dark phase and after fasting (Figure 5D–F, Suppl. Figure 4B,D), and spent more time per bout (Figure 5G). Moreover, food intake was not different after fasting or after ghrelin injection (Figure 5H,I, Suppl Figure 4C). However, in a buried food-seeking test, often used to assess olfactory capacity [39], OB^GHSR−/−^ mice took significantly longer time to find a froot loop in response to a short 4-hour fast (Figure 5K) or IP ghrelin compared to WT (Figure 5M), and while this difference was not present in the fed state (Suppl. Figure 4E) a strong trend was also observed after overnight fasting (p = 0.079; Suppl. Figure 4F). Collectively, these results suggest that the function of GHSRs in the brain is region-specific, whereby OB^GHSR^ neurons prime olfactory function to help find food and affect feeding behaviour. However, OB^GHSR^ neuronal deletion does not affect daily ad libitum food intake, or in response to fasting and IP ghrelin, which is presumably regulated by other GHSR-expressing regions including the hypothalamus and midbrain [40,41].

**Figure 6:**
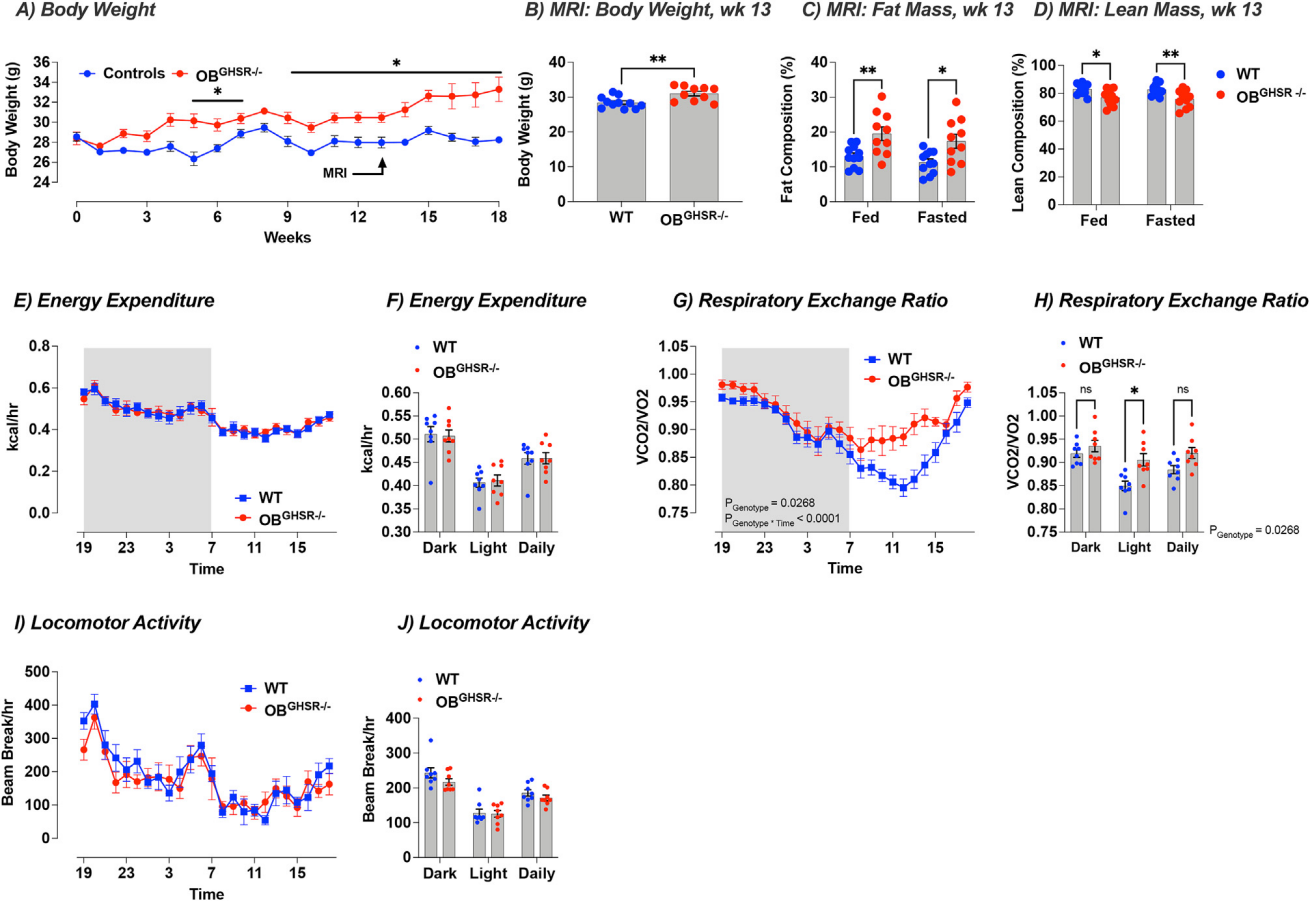
**OB**^**GHSR**^**deletion affects energy metabolism. A)** Body weight (WT = 36, OB^GHSR−/−^ = 33, 3 cohorts compiled). OB^GHSR−/−^ mice were significantly heavier (Two-way ANOVA, P_genotype_ <0.0001). **B-D)** EchoMRI body composition at 13 weeks post cre injection (WT = 11, OB^GHSR−/−^ = 10). OB^GHSR−/−^ mice exhibited a higher body weight (**B**) (two-tailed unpaired t test, P = 0.0052), with greater fat mass (**C**) and smaller lean mass (**D**) in an ad libitum or fasted state (Two-way ANOVA, P_genotype_ = 0.0002, and P_genotype_ = 0.0002 respectively). **E,F**) Energy expenditure (WT = 8, OB^GHSR−/−^ = 8). OB GHSR deletion did not change energy expenditure in either the dark or light phase (Two-way ANOVA, P_genotype_ = 0.9785). **G,H**) Respiratory exchange ratio. OB^GHSR−/−^ mice displayed an increase in the respiratory exchange ratio that was more pronounced during the light period (Two-way ANOVA, P_genotype_ = 0.0268, P_genotyoe∗time_ = 0.0002). **I,J**) Locomotor activity. OB GHSR deletion did not change locomotor activity in either the dark or light phase (Two-way ANOVA, P_genotype_ = 0.2616). Data are presented as mean +/− SEM. All specific statistical information is reported in supplementary table 1.

### 3.5 OB^GHSR^ deletion affects body weight and substrate utilisation

**Suppl. Figure 5:**
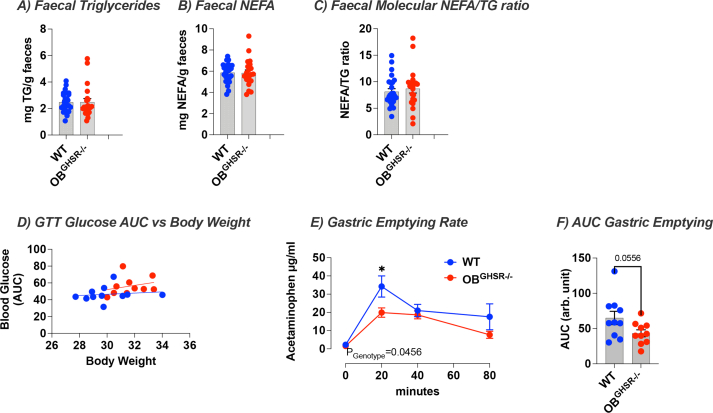
GHSR expression in the olfactory bulb affects metabolism. A) Faecal triglycerides. B) Faecal NEFA. C) Faecal NEFA/triglyceride ratio. There was no difference in the fat excretion in the faeces between the groups (WT = 25, OB^GHSR−/−^ = 21, student's t-tests). D) Correlation glucose AUC during GTT with body weight. There was no significant effect of body weight on glucose AUC during the GTT (WT = 11, OB^GHSR−/−^ = 10, Pearson r correlation). E) Gastric emptying rate during the GTT using acetaminophen as a marker for gastric emptying. F) AUC of gastric emptying rate. OB^GHSR−/−^ mice had a delayed gastric emptying (WT = 10, OB^GHSR−/−^ = 10, two-way ANOVA P_genotype_ = 0.0456, and student's t-test P = 0.0556). Data are presented as mean +/− SEM. All specific statistical information is reported in supplementary table 1.

Unexpectedly, OB^GHSR−/−^ mice were significantly heavier than control mice, with greater fat mass in the ad libitum or fasted state (Figure 6A–D). There was no difference in the fat excretion in the faeces between the groups (Suppl. Figure 5A-C), showing that differences in fat absorption cannot explain body weight gain in OB^GHSR−/−^ mice. We then placed mice in metabolic cages ∼3 weeks after OB^GHSR^ deletion, before significant weight gain, to assess whether metabolic changes were driving the increased body weight. Daily energy expenditure or the amount of energy burnt per hour depends on resting metabolic rate, the thermic effect of food intake, and the energy cost of physical activity. OB^GHSR^ deletion did not change energy expenditure in either the dark or light phase (Figure 6E,F) nor locomotor activity (Figure 6I,J). Interestingly, the absence of GHSRs in the OB increased the respiratory exchange ratio (RER), which was more pronounced in the light phase (Figure 6G,H). The RER is an estimate of macronutrient metabolism, where an RER ratio closer to 1 indicates carbohydrates being metabolized and a ratio closer to 0.7 indicates lipids being metabolized. Comparison of the RER between the two genotypes shows a significant increase in RER during the light phase in OB^GHSR−/−^ mice, suggesting that the absence of GHSR signalling in the OB shifts fuel utilization towards carbohydrates whilst promoting fat storage and driving body weight gain.

### 3.6 OB^GHSR^ deletion affects glucose metabolism

**Figure 7:**
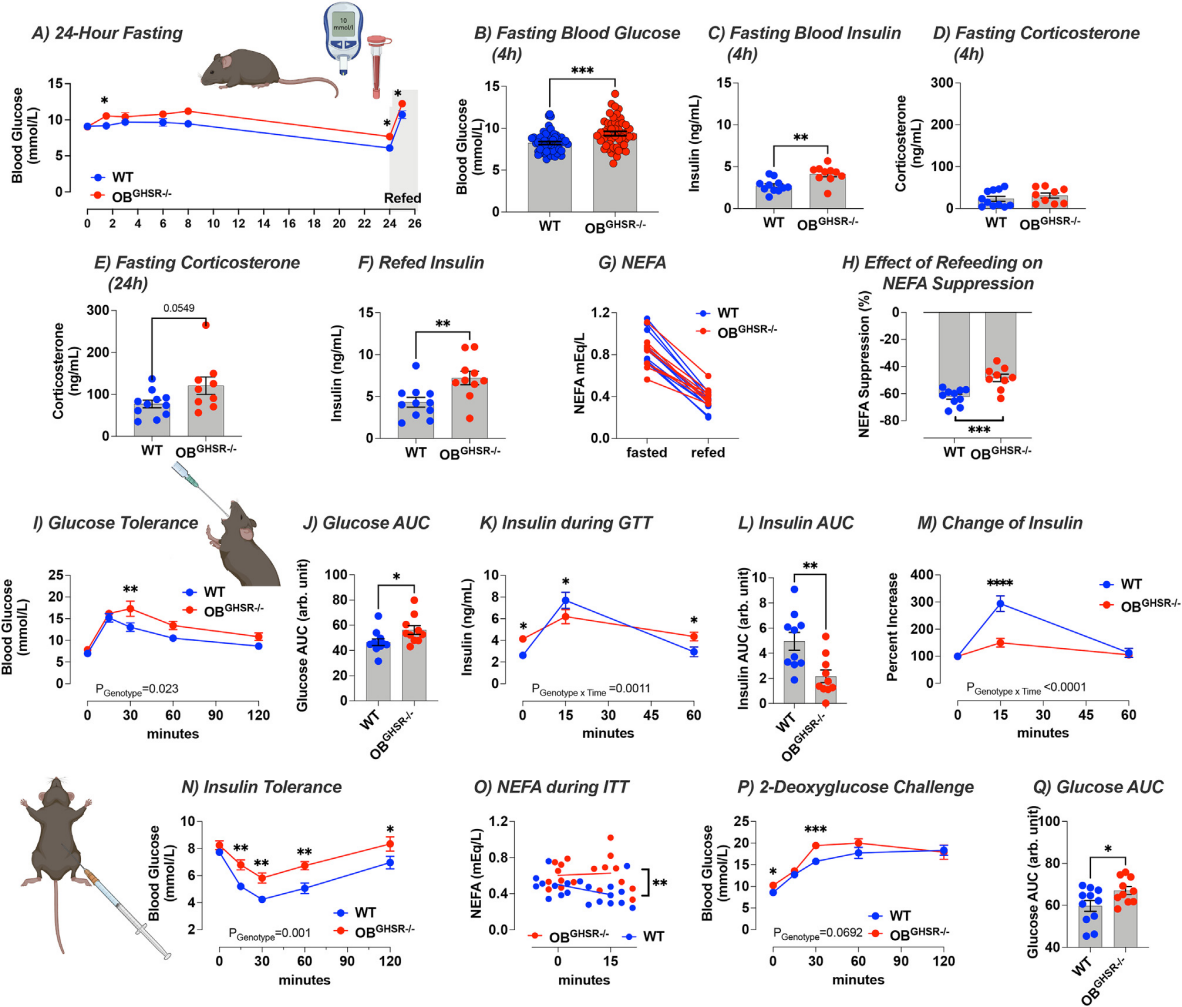
**OB**^**GHSR**^**deletion affects glucose metabolism. A-H)** 24-hour fasting time course experiment (WT = 11, OB^GHSR−/−^ = 10). Fasting increased blood glucose in OB^GHSR−/−^ mice over time (Two-way ANOVA, P_genotype_ = 0.0025). OB^GHSR−/−^ mice exhibited higher blood glucose (**B**) and blood insulin levels (**C**) after 4 h fasting (student's t-tests, P = 0.0002 and P = 0.0023). **D,E)** Blood corticosterone after 4 h (**D**), and 24 fasting (**E**) (student's t-tests, P = 0.369 and P = 0.0549). **F–H)** Refeeding with blood insulin (**F**) and non-esterified free fatty acids (**G**) after 1 h of reintroducing food. OB^GHSR−/−^ mice displayed higher blood insulin levels after refeeding (student's t-tests, P = 0.0077). There was no difference between the genotypes for non-esterified free fatty acids (NEFA) levels (Two-way ANOVA, P_metabolic state_ <0.0001). **H)** Effect of refeeding on NEFA suppression (student's t-tests, P = 0.0006). **I-M)** Glucose tolerance test (WT = 11, OB^GHSR−/−^ = 10). **I)** Blood glucose levels after an oral administration of glucose (2 g/kg). OB GHSR deletion impaired glucose clearance (Two-way ANOVA, P_genotype_ = 0.0226). **J)** Area under the curve (AUC) of glucose clearance (student's t-tests, P = 0.0361). **K)** Blood insulin levels during the glucose tolerance test (Two-way ANOVA, P_genotype∗time_ = 0.011). **L)** Insulin area under the curve (student's t-tests, P = 0.0049). **M)** Change of insulin during the glucose tolerance test (Two-way ANOVA, P_genotype∗time_ <0.0001). **N,O)** Insulin tolerance test (WT = 10, OB^GHSR−/−^ = 8). **N)** Blood glucose levels after an intraperitoneal injection of insulin (0.75 mU/g). **O)** NEFA. Compared to WT animals, insulin was less effective to lower blood glucose levels, or NEFA (Two-way ANOVA, P_genotype_ = 0.001 and P_genotype_ = 0.0067, respectively). **P,Q)** 2-Deoxyglucose challenge (WT = 11, OB^GHSR−/−^ = 10). **P)** Blood glucose levels after an intraperitoneal injection of 2-deoxyglucose (0.5 μg/g) (Two-way ANOVA, P_genotype_ = 0.0692). **Q)** AUC of glucose during 2-deoxyglucose challenge (student's t-tests). Data are presented as mean +/− SEM. All specific statistical information is reported in supplementary table 1.

In addition, OB^GHSR^ deletion resulted in impaired glucose metabolism, as OB^GHSR−/−^ mice had higher fasted glucose and insulin levels after a short fast of 4 h and during a 24-h fasting time course experiment (Figure 7A–C). Fasting corticosterone levels were not increased in OB^GHSR−/−^ mice (Figure 7D) but tended to be increased after the extended fast of 24 h (Figure 7E). Upon refeeding insulin remained elevated with an attenuated effect of refeeding to suppress non-esterified fatty acids (NEFA) (Figure 7F–H). To further evaluate glucose metabolism, we assess glucose tolerance and insulin sensitivity. After an oral administered glucose challenge (2 g/kg), glucose clearance was impaired in OB^GHSR^ deleted mice (Figure 7I,J) due to significantly reduced plasma insulin at 15 min during the oral glucose tolerance test (GTT) (Figure 7K-M). During an insulin tolerance test (ITT), insulin administration was less effective in lowering blood glucose or NEFA in OB^GHSR−/−^ mice compared to WT controls (Figure 7N-O), suggesting impaired insulin sensitivity in OB^GHSR^ deleted mice. When challenged with 2DG to assess a counterregulatory response to glucopenia, blood glucose was significantly higher in OB^GHSR−/−^ mice (Figure 7P,Q). Collectively, these studies suggest OB^GHSR^ regulate blood glucose independent from changes in body weight since GTT AUC was not correlated with body weight in either WT or KO mice (Suppl. Figure 5D). Since olfactory detection of food can impact gastric emptying as well as digestion, we examined gastric emptying during an oral GTT and observed OB^GHSR−/−^ mice had a delayed gastric emptying (Suppl. Figure 5E,F). All this suggests that OB^GHSR^ deletion impaired glucose tolerance, indicated by increased fasted blood glucose and insulin and tested by oral GTT and ITT. This was independent to differences in body weight, gastric emptying during the GTT or fat absorption (Suppl. Figure 5).

### 3.7 GHSRs are predominantly expressed on glutamate neurons in the OB and AON

**Figure 8:**
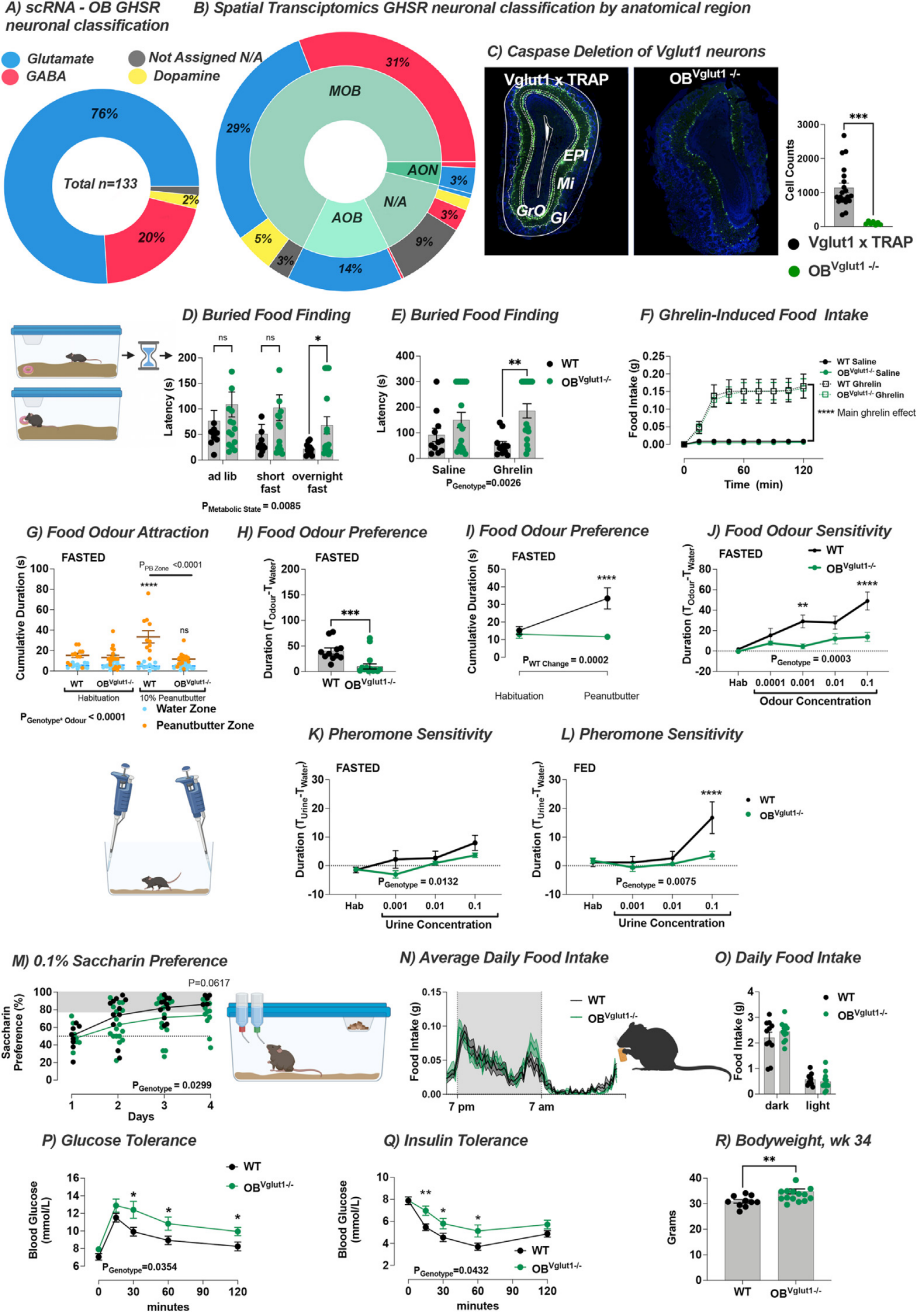
**OB**^**Vglut1**^**regulate food-seeking and glucose homeostasis.** (**A**) scRNA-seq analysis from the microdissected OB revealed 76% of GHSR-expressing neurons were glutamatergic, 20% GABAergic, 2% dopaminergic and <2% not assigned (n = 133 GHSR+). (**B**) Cross-referencing scRNA-seq and MERFISH spatial transcriptomic datasets enabled neuronal classification by anatomical structure and showed ∼ equal expression of GHSRs on glutamate and GABA neurons in the MOB, with almost exclusive GHSR expression on glutamate neurons in the AOB and AON. (C) Expression of caspase in OB^Vglut1^ neurons (OB^Vglut1−/-^) caused significant cell loss (WT = 20, OB^Vglut1 −/−^ = 7). (**D**) Buried food finding was significantly delayed in overnight fasted OB^Vglut1−/−^mice or in response to ghrelin injection (**E**) with no effect on ghrelin-induced food intake (**F**) WT = 11, OB^Vglut1 −/−^ = 17. (**G**) In an odour attraction test, fasted OB^Vglut1−/-^ mice spent significantly less time in the zone with 10% peanut butter (PB), leading to less food odour preference (**H, I**) compared to WT mice (WT = 11, OB^Vglut1 −/−^ = 17). (**J**) The sensitivity to diluted peanut butter (PB) solution was significantly impaired in fasted OB^Vglut1−/-^ mice compared to WT (WT = 11, OB^Vglut1 −/−^ = 17), whereas fed OB^Vglut1−/-^ mice were less sensitive to urine diluted in water than WT mice (**K, L**) (WT = 11, OB^Vglut1 −/−^ = 17). (**M**) OB^Vglut1−/-^ mice displayed significantly lower saccharin preference (main effect of genotype) compared to WT mice (WT = 11, OB^Vglut1 −/−^ = 17) but no difference in average daily food intake (**N**, **O**) (WT = 11, OB^Vglut1 −/−^ = 12). Glucose clearance was slower in OB^Vglut1−/-^ mice during an oral glucose tolerance test (**P**) and the insulin-induced suppression of blood glucose was not as great as in WT mice during an insulin tolerance test (O) indicating OB^Vglut1−/-^ mice had impaired glucose handling and were less sensitive to insulin. (R) Body weight was significantly higher in OB^Vglut1−/-^ mice at the end of the experiment. Data are presented as mean +/− SEM. All specific statistical information is reported in supplementary table.

**Suppl. Figure 6:**
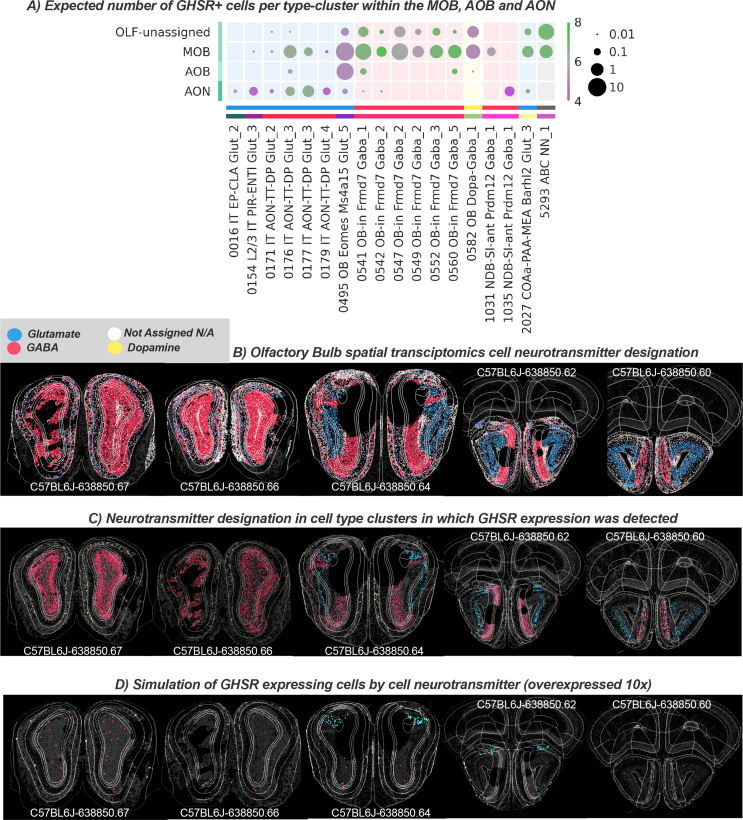
scRNA-seq and spatial transcriptomic classification of GHSR + cells in the MOB, AOB and AON. A) The expected number of GHSR + cells from different cell clusters within the MOB, AOB and AON. The dot size reflects the number, whereas shaded blue squares designated glutamatergic clusters, the shaded red squares reflect GABAergic cluster and the yellow shared squares highlight a dopamine cluster. B) Images show the olfactory bulb sections on which spatial transcriptomics was performed. Colours indicate the location of Glutamate, GABA, Dopamine and not assigned neurons according to spatial transcriptomic data. Black areas within sections of the OB indicated a lack of data for that region, as seen most predominantly in sections 638850.67, 638850.64 and 638850.62. C) Images show the location and neurotransmitter designation (Glutamate, GABA, dopamine, N/A) from spatial transcriptomic analysis in which GHSR + cells were detected. D) Images depict the location and neurotransmitter designation of GHSR + cells expressed 10× above the threshold limit.

The OB is made of multiple neuronal types of which the majority are mitral and tufted glutamatergic neurons or GABAergic interneurons in the mitral, external plexiform and granular cell layers respectively [21]. The OB also receives significant inputs from the nearby AON as a part of the primary olfactory cortex, an area where we observed viral expression of cre recombinase and cre-mediated expression of RFP. To ascertain the neuronal subtypes containing GHSR expression, we used a bioinformatics approach from a high-resolution single cell and spatial transcriptomic analysis of the OB [34]. Single cell RNA sequencing (scRNA-seq) analysis identified 133 GHSR+ cells of which 76% were classified in glutamatergic type-clusters, 20% in GABAergic type clusters, 2% in dopamine type clusters and >2% unassigned (Figure 8A; Suppl. Table 1). By combining both scRNA-seq with spatial transcriptomic datasets, we created the spatial cell-type atlas for GHSR cells in the OB and AON. This approach identified the almost exclusive expression of GHSRs on glutamatergic neurons in the AOB (Figure 8B; Suppl. Figure 6), consistent with the EGFP expression in the OB from *Ghsr*-p2A-cre mice crossed with fsTRAP mice (Suppl. Figure 1). Within the MOB, there were an equal number of glutamatergic and GABAergic neurons expressing GHSRs, and cells in the AON expressing GHSRs were predominantly glutamatergic (Figure 8B), although some limitations with the spatial transcriptomic dataset include limited section number, section damage causing minor misalignment (Suppl. Figure 6).

### 3.8 OB^Vglut1^ regulate food-seeking and glucose homeostasis

**Suppl. Figure 7:**
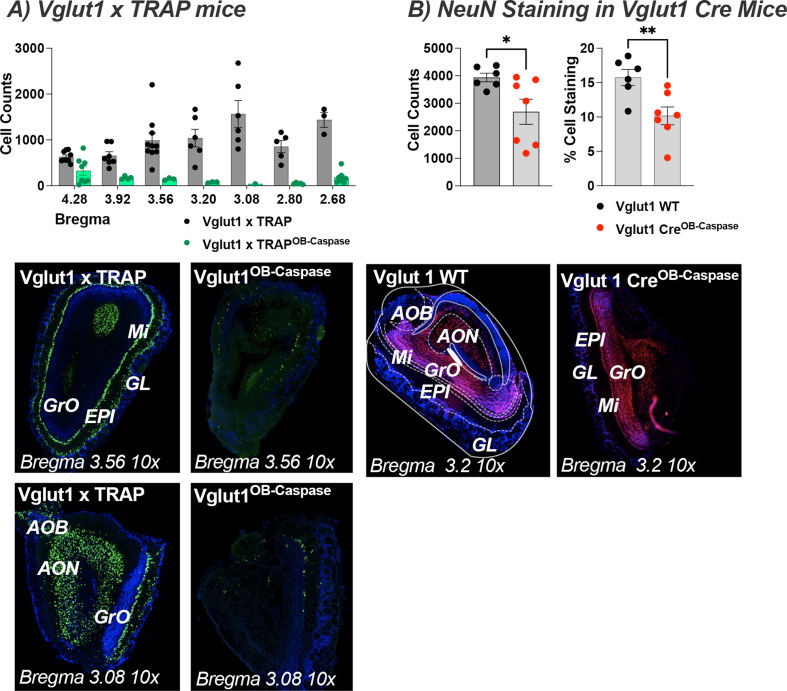
Validation of OB^Vglut1^ neuronal ablation. A) Vglut1 GFP neurons were counted from control mice without caspase ablation and from OB^Vglut1−/-^ mice with caspase ablation. WT mice were *Vglut1*-ires-cre mice crossed with cre-dependent fsTRAP-EGFP-L10a (Vglut1 x TRAP) and OB^Vglut1−/−^ mice were Vglut1 x TRAP mice injected with AAV-FLEX-caspase into the OB (Vglut1^OB−Caspase^). There were significantly fewer Vglut1 GFP neurons in all sections from bregma 4.28 to bregma 2.68 in Vglut1^OB−Caspase^ compared to control Vglut1 x TRAP mice. B) NeuN staining was also significantly lower in the OB in Vglut1^OB−Caspase^ compared to WT mice. Data are presented as mean +/− SEM. All specific statistical information is reported in supplementary table 1.

**Suppl. Figure 8:**
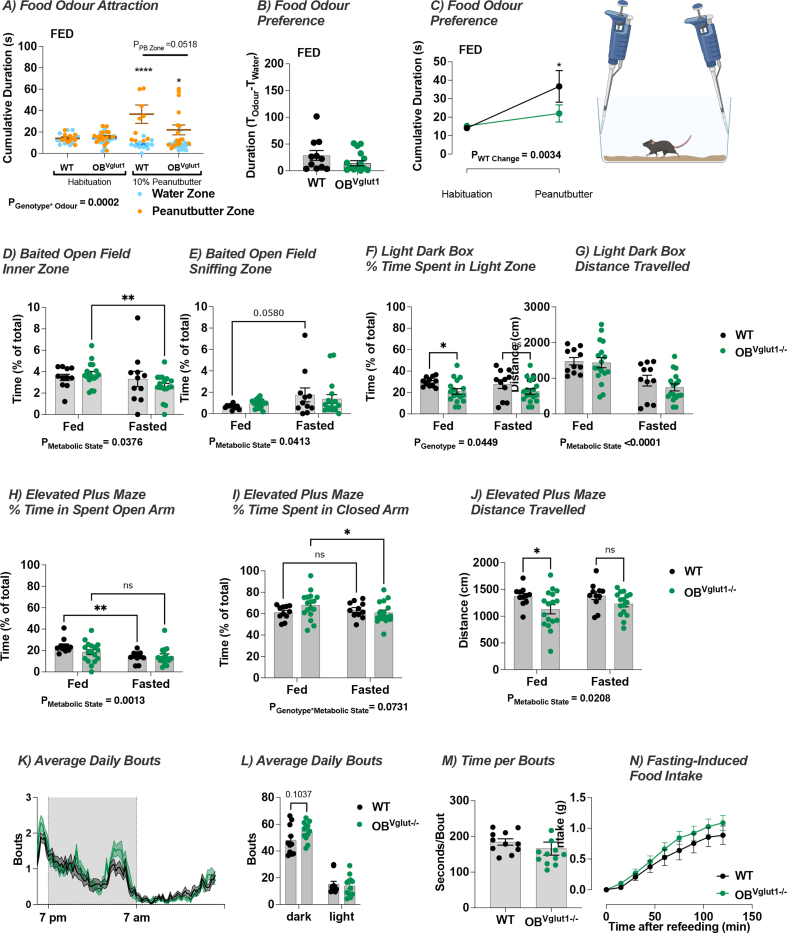
Behavioural and feeding effects from OB^Vglut1^ neuronal ablation. A) Food odour attraction in the ad libitum fed state with odour preference duration (B, C). Time spent in the inner zone (D) and sniffing zone (E) of the baited open field or light zone of the of the light dark box (F). Distance moved within the light zone of the light dark box (G). Time spent with the open arm (H), closed arm (I) and distance travelled (J) within the elevated plus maze. Average daily feeding bouts (K, L), bout duration (M) and refeeding after fasting (N). Data are presented as mean +/− SEM. All specific statistical information is reported in supplementary table 1.

As the scRNA-seq dataset showed 76% of GHSR-expressing OB cells are glutamatergic, we temporally ablated OB^Vglut1^ neurons in adult *Vglut1*-ires-cre mice using AAV-FLEX-taCasp3-TEVp [42] (OB^Vglut1−/−^). Analysis of GFP labelled OB^Vglut1^ neurons in Vglut1 x TRAP mice and NeuN staining in Vglut1-ires-cre mice revealed successful ablation of Vglut1 neurons in the MOB, AOB and AON (Figure 8C; Suppl. Fig 7). OB^Vglut1^ neuronal ablation caused a delay in buried food finding after an overnight fast and in response to ghrelin injection without a difference in ghrelin-induced food intake, similar to that seen in OB^GHSR−/−^ mice (Figure 8D–F). The ablation of OB^Vglut1^ neurons significantly impaired olfactory function in odor preference and sensitivity tests. Fasted WT mice showed a strong attraction to 10% peanut butter solution and were more sensitive to odor dilutions when compared to OB^Vglut1−/−^ mice (Figure 8G–J; Suppl. Fig 8A-C). WT mice were more sensitive to urine solutions in the fed state, which was significantly lower in OB^Vglut1−/−^ mice (Figure 8K-L). Behavioral analysis revealed a significantly lower saccharin preference in OB^Vglut1−/−^ mice (Figure 8M), although anxiety-related tests highlighted a mild anxiety-like phenotype with only a significant effect of genotype observed in the light dark box, but not the EPM or baited open field (Suppl Figure 8D-J). No differences in daily food intake, feeding bouts, bout duration or fasting-induced feeding were observed (Figure 8N-O; Suppl Figure 8K-N). Similar to OB^GHSR−/−^ mice, ablation of OB^Vglut1^ neurons significantly impaired glucose clearance after oral glucose gavage as well as insulin sensitivity by ITT, and body weight was significantly higher at the end of the experimental period (Figure 8P-R).

## 4 Discussion

Food odors are important sensory cues that convey environmental information about food availability, palatability, and calorie content to an organism. These cues invigorate food-seeking, spatial navigation, taste and reward value processing [9], as well provide preparatory signals to prime the appropriate metabolic response to incoming nutrients [1,6]. With this in mind, it is not surprising that hunger increases olfactory sensitivity [5,[17], [18], [19]]. Here we examined the hypothesis that OB^GHSR^ expression is a hunger-signaling mechanism that links metabolic state to olfaction and regulates the behavioral and metabolic consequences of heightened olfactory function. Indeed, OB^GHSR^ deletion decreased olfactory sensitivity to food and non-food odors in various olfactory performance tasks in both fed and fasted conditions. Although daily total food intake or ghrelin-induced food intake were not different, OB^GHSR^ deletion decreased the number of feeding bouts in the dark phase and impaired food finding in a buried food-seeking test. Surprisingly, OB^GHSR^ deletion increased body weight, fat mass accumulation by reducing lipid utilisation and impaired glucose tolerance. Moreover, OB^GHSR^ deletion increased anxiety-like behavior and anhedonia while reducing exploratory behavior in novel environments, particularly in the fasted state. OB^GHSRs^ are predominantly expressed on glutamate neurons in the MOB, AOB and AON and subsequent ablation of OB^Vglut1^ neurons reduced ghrelin-increased buried food finding and phenocopied the behavioral and metabolic effects of OB^GHSR^ deletion. Our results demonstrate OB^GHSRs^, most likely on OB^Vglut1^ neurons, influence olfactory sensitivity, anxiety-like, exploratory, feeding behavior (not food consumption) and peripheral energy and glucose metabolism.

The mechanisms underlying the behavioral and metabolic phenotypes caused by OB^GHSR^ deletion remain unknown. However, it may involve altered G-protein coupled signaling pathways since GHSRs possess highly promiscuous signalling capacity through various pathways including Gα_q/11_, Gα_12_, Gi/o, G13 and arrestin [[43], [44], [45]]. Moreover, GHSRs display high constitutive activity, which can regulate downstream signaling in the absence of ghrelin binding [46] and influence intracellular trafficking and synaptic transmission [47,48]. Indeed, the constitutive activity of GHSRs regulates both behavioural and metabolic outcomes [49,50].

Our results suggest that the deletion of OB^GHSRs^ suppresses OB output to increase anxiety-like and reduce exploratory behavior. Indeed, removal of OB output via bulbectomy causes anxiety-like and depressive-like symptoms [10] and reduced neurogenesis in the OB is associated with depressive-like behavior [51,52]. These studies suggest that the suppressed neural activity from the OB after GHSR deletion mediates the anxiety-like and depressive-like behavior. Interestingly, we observed mild anxiety-like and depressive-like effects of OB^Vglut1^ neurons, as genotype differences were only observed in the light dark box and two bottle choice assays. This was less pronounced compared to OB^GHSRs^ deletion, suggesting other OB neurons may also mediate the behavioural effects of OB^GHSRs^ deletion, although future studies are required to test this idea.

Even though the OB has significant GHSR expression [25,27,33], and the highest uptake of ghrelin in the entire brain [29], this is the first study to examine the functional role of GHSRs in the OB. Of note, OB^GHSR^ deletion significantly impaired olfactory sensitivity to palatable food (peanut butter, froot loop), non-food odors (rosewater) and social (urine) odors, as judged by olfactory habituation and olfactory preference tests. Although olfactory function was impaired in both fed and fasted mice after OB^GHSR^ deletion, fasting was more often associated with greater interest in food odors, such as time spent sniffing froot loops vs rose water and time spent sniffing peanut butter-scented open field. Collectively, results are consistent with the idea that fasting increases sensitivity to food odors [19,[53], [54], [55]], an effect consistently seen across species including humans, mice and flies, and that hunger dampens rival motivations to prioritise food seeking [56,57]. Moreover, many of the strongest anxiety-like behavioral and food-seeking effects observed after OB^GHSR^ deletion occurred in fasted mice. Indeed, the metabolic functions of the ghrelin system are potentiated in states of metabolic need, such as fasting, and attenuated in states of metabolic excess, culminating in ghrelin resistance in diet-induced obese mice [[58], [59], [60], [61]]. These observations have led to the classification of ghrelin as a survival hormone [23].

The impact of OB^GHSR^ deletion on anxiety-like and anhedonia mirrors the effects of impaired olfactory function on depression and anxiety in humans [62] and mice [63], as well as the loss of pleasure and enjoyment of food in anosmic humans [8,9]. These findings all underscore the use of olfactory bulbectomy as a rodent model of depression-like behavior [10]. Interestingly, ghrelin and GHSR function are also linked with reduced anxiety-like and depressive-like behavior [[64], [65], [66], [67]], including following olfactory bulbectomy in mice [68], although the main sites of action have not been fully elucidated. Previous studies suggested the exaggerated depressive-like symptoms associated with *Ghsr* deletion in mice could be overcome by re-expressing GHSRs selectively in catecholaminergic neurons [69], by overexpressing the GHSR in the amygdala [70] or by augmenting hippocampal neurogenesis [71], implicating the ventral tegmental area, amygdala and the hippocampus. We did not examine anxiety-like or depressive-like behaviours after IP ghrelin administration in OB^GHSR^ deleted mice, as IP ghrelin has the potential to act on these different brain regions. Our studies, however, focussed on the GHSR and highlight an important and unappreciated physiological role for OB^GHSR^ signalling to alleviate anxiety-like and depressive-like behaviors, as well as enhance exploratory behavior, particularly in the fasted state. Intriguingly, a similar effect was previously reported in GHSR KO mice, where these mice exhibited deficits in anxiolytic-like behaviour only in the energy-restricted state [66]. Therefore, our results suggest OB^GHSRs^ play a specific anxiolytic role to encourage exploration and optimal food-seeking and foraging behaviour.

OB^GHSR^ deletion did not affect daily cumulative or daily average food consumption, nor did it affect refeeding after fasting or in response to IP ghrelin. These results are not surprising considering GHSR expression remained intact elsewhere in the brain and body. For example, several studies have shown crucial roles for GHSRs on AgRP neurons or TH neurons in the neural control of food intake [40,41,69,72]. Nevertheless, GHSRs in the OB were crucial for food-seeking in a buried-food finding test in short-term fasting, but not fed mice, and in response to IP ghrelin. Moreover, OB^Vglut1^ neuronal ablation delayed buried-food finding in response to ghrelin injection, suggesting an important role for GHSR expression on OB^Vglut1^ neurons. This is supported by scRNA-seq data showing 76% expression of GHSRs on OB^Vglut1^ neurons.

In addition, feeding behavior as assessed by bout number and duration, was significantly impaired after OB^GHSR^ deletion. Given the impact of OB^GHSR^ deletion on anxiety-like and exploratory behavior, we argue that this behavioral phenotype is likely a contributing factor to the impaired food-seeking and feeding behavior observed. Moreover, this supports the hypothesis that OB^GHSRs^ are required to optimise foraging by facilitating the appropriate behavioral adaptations to low food availability. The lack of effect of OB^Vglut1^ neuronal ablation on feeding behavior suggests the actions of OB^GHSRs^ on feeding behaviour are mediated by other non-glutamatergic populations.

Hunger-sensing AgRP neurons enhance food odor attraction over social odors, as well as promote food intake [54]. Although GHSRs are co-expressed on >90% of NPY/AgRP neurons in the ARC [73,74], our discovery demonstrates a specific role for GHSRs in the OB, independent from AgRP neurons in foraging and anxiety-like behavior but not food consumption. Olfaction is usually the first sensory modality predicting food characteristics and plays an important role in food-seeking for many animals [4], and olfactory detection of food odors can acutely suppress AgRP neuronal activity [54]. Thus, olfaction is likely to relay external sensory information of energy availability to AgRP neurons to facilitate this integration. Our data predicts the coordinated action of GHSR-dependent hunger-signaling in the OB, together with hunger sensing in hypothalamic AgRP neurons, is required for appropriate behavioral and metabolic response to fasting and low energy availability, although further studies are required to explore this interaction.

While total caloric consumption was not affected by OB^GHSR^ deletion, we observed an increase in body mass and fat mass. This is somewhat surprising given that 1) whole-body deletion of the GHSR results in reduced body weight after HFD feeding [75], 2) ghrelin deletion protects against early onset of obesity [76] and 3) rebound weight gain after diet-induced weight loss [59], although not all knockout studies have reported similar findings [77,78]. However, olfactory dysfunction is associated with weight gain and obesity [[79], [80], [81]] and increased olfactory sensitivity prevented diet-induced obesity in both genetic and pharmacological models [[14], [15], [16]]. These studies suggest that weight gain after OB^GHSR^ deletion may be a secondary consequence caused by impaired olfactory sensitivity, an observation supported by the impaired olfactory sensitivity and weight gain seen after OB^Vglut1^ neuronal ablation. The regulation of peripheral lipid utilisation is a likely link since exposure to a food odor triggers fat mobilisation and utilisation in mice [6] and worms [3]. Consistent with this, we observed less fat utilisation and greater carbohydrate utilisation after OB^GHSR^ deletion. Notably, differences in nutrient partitioning are associated with perturbations in body weight gain [82].

Deletion of GHSR signaling in the OB also impacted glucose metabolism. OB^GHSR−/−^ mice had higher fasted blood glucose and a limited ability to clear glucose due to impaired insulin secretion, reduced insulin sensitivity and increased glucose production when challenged with 2-deoxyglucose. Moreover, ablation of OB^Vglut1^ neurons also impaired glucose clearance and insulin sensitivity, further reinforcing the important role of normal olfaction on blood glucose regulation. Indeed, olfactory dysfunction is linked to impaired glucose regulation and type 2 diabetes [83], in line with our findings. Nevertheless, our results are in contrast with the observation that conditional ablation of olfactory sensory neurons in the olfactory epithelium prevented insulin resistance and improved glucose clearance [13].

Our results shed light on the role of the OB as an integrator of metabolic state and suggest an important neuroendocrine role for the OB. This idea is supported by the expression of various metabolic hormone receptors, including insulin, leptin, cholecystokinin, orexins, and ghrelin [21]. We hypothesize that olfactory sensitivity is an important sensory integrator of relevant olfactory cues predicting food availability and calorie content. In response to the olfactory detection of known foods or food cues, pre-emptive metabolic and behavioral responses are engaged to facilitate foraging as well as preparation for an incoming meal. This view is informed by the observation that the sensory detection of food or food odors primes hepatic metabolic gene expression and lipid metabolism [1].

In summary, we show that selective GHSR deletion in the OB suppresses normal olfactory function, decreases odor-based food-seeking, and exploratory locomotion and leads to anxiety- and depressive-like behaviors. Moreover, OB^GHSR^ deletion increased body weight gain by restricting fatty acid substrate utilisation and impaired glucose metabolism. Although we observed altered feeding behavior, OB^GHSR^ deletion did not affect food consumption. Moreover, GHSRs are mostly expressed on glutamatergic neurons in the OB and OB^Vglut1^ neuron ablation replicates some of the effects of OB^GHSR^ deletion, including olfactory dysfunction, delaying buried-food finding, impaired glucose tolerance and insulin sensitivity. Finally, we only used male mice in these studies, and as such, an important limitation of this study of this study is the inability to assess sex differences.

Collectively, we suggest OB^GHSRs^ maintain olfactory sensitivity, leading to several behavioral and metabolic adaptations to help an organism respond to low energy availability. Intact OB^GHSR^ signaling confers appropriate behavioral resilience to explore and exploit foraging opportunities in a potentially anxiogenic environment. At the same time, OB^GHSR^ signalling primes olfactory-driven metabolic responses, including glucose and lipid regulation. An understanding of the precise OB^GHSR^ neural circuits is an important avenue for future research, especially considering the unique therapeutic potential of intranasal delivery to target pharmacological treatments for metabolic disorders.

## Disclosure statement

J.M.Z. receives research funding from Novo Nordisk for another project and consulted for Helsinn Healthcare S.A. and Dexcel Pharma Technologies Ltd. during the time these studies were performed. The other authors have nothing to disclose.

## CRediT authorship contribution statement

**Romana Stark:** Writing – review & editing, Writing – original draft, Methodology, Investigation, Formal analysis, Data curation, Conceptualization. **Harry Dempsey:** Software, Investigation, Formal analysis. **Elizabeth Kleeman:** Investigation. **Martina Sassi:** Investigation. **Sherri Osborne-Lawrence:** Investigation. **Sepideh Sheybani-Deloui:** Investigation. **Karl Austin-Muttitt:** Methodology, Investigation. **Jonathan Mullins:** Supervision. **Jeffrey M. Zigman:** Writing – review & editing, Resources. **Jeffrey S. Davies:** Writing – review & editing, Validation, Supervision, Methodology, Investigation. **Zane B. Andrews:** Writing – review & editing, Writing – original draft, Validation, Supervision, Software, Resources, Project administration, Methodology, Investigation, Funding acquisition, Conceptualization.

## Declaration of competing interest

J.M.Z. receives research funding from Novo Nordisk for another project and consulted for Helsinn Healthcare S.A. and Dexcel Pharma Technologies Ltd. during the time these studies were performed. The other authors have nothing to disclose.

All other authors report no conflict of interest.

## Data Availability

Data will be made available on request.
